# Leadership’s long arm: The positive influence of digital leadership on managing technology-driven change over a strengthened service innovation capacity

**DOI:** 10.3389/fpsyg.2023.988808

**Published:** 2023-02-01

**Authors:** Timo J. J. Brunner, Tobias Schuster, Claudia Lehmann

**Affiliations:** LF Group Chair for Digital Innovation in the Service Industries, HHL Leipzig Graduate School of Management, Leipzig, Germany

**Keywords:** digital leadership, digital transformation, technology-driven change, service innovation, dynamic service innovation capabilities, dynamic capabilities

## Abstract

**Introduction:**

In this qualitative study, we examine digital leadership (DL) capabilities and their positive influence on the management of technology-driven change by leveraging service innovations. The context of digital transformation (DT) has triggered a new leadership paradigm, among others referred to as digital leadership (DL). However, despite its practical relevance, leadership research has yet paid little attention to conceptualise DL as an approach to digitally transform organisations.

**Methods:**

Drawing on mid- and top-level mangers’ experiences with service innovation projects, and based on Grounded Theory, we develop a taxonomy of DL-related capabilities and a conceptual framework which exemplifies their influences on dynamic service innovation capabilities (DSICs). DSICs build on the dynamic capabilities view (DCV) and represent the “organisational muscle” to repeatedly deliver service innovations indicating an effective management of technology-driven change.

**Results and Discussion:**

Taxonomy results show that aggregated dimensions in terms of a digital leader’s personal, social, and organisational capital serve as underpinnings (DL-related capabilities) to drive strategic change in DT contexts. The conceptual framework further reveals that especially the personal and organisational capital of a digital leader owns several strong and moderate influences on DSICs which demonstrates DL’s “long arm” on the management of technology-driven change. Our findings contribute to leadership research by advancing the conceptualisation of DL and by adding a novel micro-foundational perspective towards the DCV discourse. As organisations struggle to realise the full benefits of DT initiatives, our results also provide a valuable contribution for practitioners by supporting them to strategically prepare for the human-related challenges of DT.

## 1. Introduction

The rapid progress of technological change confronts today’s organisations with significant changes such as new competition mechanisms, organisational structures, and work design. This goes in line with the current age of ubiquitous computing characterised by organisations which are increasingly equipped with information and communication technology and a fusion of the digital and physical world (Cascio and Montealegre, 2016; Schwarzmüller et al., 2018). This development suggests that digital transformation (DT) as a major driver of organisational change represents a prime topic for firms around the world (Fitzgerald et al., 2014; Kane et al., 2015; Kaufman and Horton, 2015; von Leipzig et al., 2017). DT is understood as the existence of profound changes for states, society, and organisations due to the adoption of modern technologies such as Cloud Computing, Artificial Intelligence, Internet of Things, Big Data, digital platforms, and social networking (Reis et al., 2018). The ongoing COVID-19 pandemic is further fuelling these changes by forcing organisations to rapidly move to digital working models and to adjust their business models to the new conditions (Kudyba, 2020; Priyono et al., 2020). Microsoft CEO Satya Nadella summarises these developments with *“we have seen two years of digital transformation in two months”* (Spataro, 2020). Despite the “new normal” of technology-driven change (Carroll and Conboy, 2020), studies of successful organisations indicate that DT less depends on the adoption of technology but on leadership and its strategies (Westerman and McAfee, 2012; Kane et al., 2015). As observed by Harvard Business Review Analytic Service Report (2017), leaders’ role in driving positive results from investments in digital technologies has increased in the past years. In this context, leaders take a pivotal role for a successful DT journey of their organisation (Hunt, 2015), such as by establishing suitable organisational structures and processes, and by fostering a positive outlook towards digitalisation at the employee side (Wipulanusat et al., 2017). However, research also revealed that DT is still frequently considered as a technology rather than a human-centric approach of change (Frankiewicz and Chamorro-Premuzic, 2020; Philip, 2021). This often leads to an underrepresentation of the leadership role resulting in incomplete DT initiatives that negatively affect business performance (Kotter, 2009; Deloitte, 2017; Davenport and Westerman, 2018).

In other words, although the topic of DT is prominent on leadership agendas (A. Singh and Hess, 2020), there is still limited research in terms of studies for strategic change focusing on how organisations can be digitally transformed (Warner and Wäger, 2019). In this vein, to cover the leadership dimension of DT, literature has among others coined the term of digital leadership (DL; Fisk, 2002; Westerman and McAfee, 2012; El Sawy et al., 2016; Buvat et al., 2018; Kane et al., 2019; Klus and Müller, 2021; Benitez et al., 2022). According to Eberl and Drews (2021), DL can be defined as *“a complex construct aiming for a customer-centered, digitally enabled, leading-edge business model by (1) transforming the role, skills, and style of the digital leader, (2) realizing a digital organization, including governance, vision, values, structure, culture, and decision processes, and (3) adjusting people management, virtual teams, knowledge, and communication and collaboration on the individual level.”* This definition demonstrates that, instead of optimisations of the present organisational state (management perspective; Kotter, 2000), DL aims for fundamental changes of business conditions on multiple levels to achieve sustainable competitiveness (Kane et al., 2015; El Sawy et al., 2016). DL further differentiates from the traditional leadership perspective in the objective of developing a digital strategy and a culture that enables an organisation to digitally transform in a business ecosystem (Kane et al., 2015; El Sawy et al., 2016). For digital leaders, this requires a blending of traditional and new leadership capabilities such as a transformative vision and forward-looking perspective, digital literacy, and adaptability (Schwarzmüller et al., 2018; Kane et al., 2019; Petry, 2019; Klus and Müller, 2021). Although DL owns a strong practical relevance, it is weakly conceptualised in the current academic discourse indicated in aspects such as the definitional fuzziness of the term, e.g., in terms of a missing differentiation between e-leadership and DL (Eberl and Drews, 2021). This goes in line with the fragmented character of DL studies across a variety of research disciplines (Cortellazzo et al., 2019; Gierlich-Joas et al., 2020). Overall, DL is yet underrepresented in leadership research streams (Schwarzmüller et al., 2018; Cortellazzo et al., 2019; Erhan et al., 2022). As an example, the latest review of leadership research published in The Leadership Quarterly (LQ) displays e-leadership in only 0.2% of the LQ publications between 2010 and 2019, whereas DL does not appear as a focal leadership theory (Gardner et al., 2020).

We conclude that the relationship between DL-related capabilities and the organisational ability to manage technology-driven change has received little research attention so far provoking the following research question: *How do DL-related capabilities influence the management of technology-driven change by leveraging service innovations?*

We aim to answer our research question in a service innovation context which we reason in three respects. First, service innovations are pervasive in today’s industries and markets (Gustafsson et al., 2020; Verma and Gustafsson, 2020; Witell et al., 2020) and their positive influence on economic growth is increasingly being recognised (Aksoy et al., 2020; Khanra et al., 2021; Ostrom et al., 2021). Second, service innovations are often subject to DT initiatives in service and manufacturing firms (Mattsson and Andersson, 2019; Soto Setzke et al., 2021; Wang, 2022). This means that service innovations play a vital role in coping with organisational challenges related to DT (Schoemaker et al., 2018; Singh et al., 2020; Singh and Hess, 2020). Third, leadership can positively influence the service innovation capacity (Kao et al., 2015; Anning-Dorson, 2016; Chiu, 2018; Urquhart et al., 2018; Karatepe et al., 2019), however in the context of DL, this influence remains a research gap. To gather insights about how to manage organisational change in a service innovation context, we apply the framework of dynamic service innovation capabilities (DSICs; den Hertog et al., 2010). As an established (Babaei and Aghdassi, 2022; Zhang et al., 2022) and validated (Janssen et al., 2016) framework in service research, which is grounded in the dynamic capabilities view (DCV; Eisenhardt and Martin, 2000), DSICs represent organisational antecedents to repeatedly exploit service innovations for a sustained competitive advantage. They include signalling user needs and technological options, conceptualising (un-)bundling, co-producing and orchestrating, scaling and stretching, and learning and adapting (den Hertog et al., 2010). We propose that the DSICs framework is particularly useful in the context of our research question since it provides an operationalisation of how to effectively manage DT. We therefore utilise this framework to examine influences of DL-related capabilities on the ability to deal with challenges of DT. Based on the above-stated research question our study delivers two outcomes, a taxonomy of DL-related capabilities along different dimensions, and a conceptual framework illustrating their influences on DSICs. For this purpose, we apply a qualitative research methodology which includes an analysis of 39 semi-structured expert interviews conducted in two rounds with top- and mid-level managers at German service and manufacturing firms. Research insights are gathered with the help of Grounded Theory (Corbin and Strauss, 1990; Gioia et al., 2013).

Our work contributes both to theoretical and managerial outlets. From a theoretical perspective, this study extends the body of knowledge of the so far weakly conceptualised phenomenon of DL (Schwarzmüller et al., 2018; Eberl and Drews, 2021). We achieve this progress by offering a holistic and integrated view of DL-related capabilities in terms of a digital leader’s personal, social, and organisational capital promoting a uniform understanding of DL. Our study also contributes to the DCV by adding a new perspective to microfoundations (skills, processes, routines, organisational structures, decision rules, and disciplines) of dynamic capabilities (Teece, 2007). This is realised by the chosen service innovation context, in which we consider the intersection between DL-related capabilities and DSICs to drive technology-driven change, and where we especially illustrate the influence of a digital leader’s personal and organisational capital to repeatedly deliver service innovations. From a managerial perspective, the results of this study are valuable for organisations still struggling to realise full benefits of DT initiatives (Fitzgerald et al., 2014). In this sense, the study sheds light on relevant DL-related capabilities in DT contexts and therefore is valuable as a tool for leadership selection and development (Cascio and Aguinis, 2008), addresses change in leadership understandings towards more digital ones (Fisk, 2002; Patterson et al., 2005; Petry, 2019), and pinpoints determinants to manage service innovations to drive DT in organisations (Denti and Hemlin, 2012; Zhu et al., 2022).

## 2. Theoretical background

### 2.1. Conceptualising digital leadership

To achieve strategic change in DT contexts, literature has among others derived the concept of DL (Zulu and Khosrowshahi, 2021). The demand for DL emerges as today’s organisations increasingly transform into digital workplaces understood as *“the physical, cultural and digital arrangements that simplify working life in complex, dynamic and often unstructured working environments”* (Dery et al., 2017, p: 136) which require leaders with different mindsets, skills, and behaviours to ensure the competitiveness of their organisation in digital spheres (Erhan et al., 2022). However, despite its practical relevance, extant research on DL shows a weakly conceptualised picture, prominently mentioned by Benitez et al. (2022, p: 2): “*Just a few prior studies have conceptualized and analyzed what digital leadership and/or digital leader mean […]. However, in reality, digital leadership capability is essential to enable digital transformation, and despite its potential to create business value, digital leadership capability is scarce among contemporary firms*.” This conceptualisation issue can especially be reasoned with the different contexts and disciplines the phenomenon is studied (Franco, 2020) resulting in issues such as definitional fuzziness (Eberl and Drews, 2021). The lacking differentiation between e-leadership (Avolio et al., 2000, 2014) and digital leadership (El Sawy et al., 2016) denotes a relevant indicator for the “fuzziness issue.” In this sense, compared to e-leadership, which is grounded in Adaptive Structuration Theory (Avolio et al., 2000), current literature on digital leadership lacks a common theoretical foundation. To illustrate the breadth of understanding regarding DL, we curated an overview of DL definitions as shown in Table 1.

**Table 1:** Overview of DL definitions.

Authors	Area	Type	Approach	Definition
Kane et al. (2019)	Information Systems research	Research paper	Empirical	A combination of traditional and new leadership skills to cope with challenges of digital transformation such as increased pace of business, cultural shifts, distributed workplaces, higher expectations of productivity
Benitez et al. (2022)	Information Systems research	Research paper	Empirical	“Portfolio of digital, market, business, and strategic leadership skills to lead and manage inter-disciplinary teams to drive the digital transformation of the firm.”
El Sawy et al. (2016)	Information Systems research	Research paper	Empirical	“Doing the right things for the strategic success of digitalization for the enterprise and its business ecosystem. Digital leadership means thinking differently about business strategy, business models, the IT function, enterprise platforms, mindsets and skill sets, and the workplace.”
Eberl and Drews (2021)	Information Systems research	Conference paper	Theoretical	“A complex construct aiming for a customer-centered, digitally enabled, leading-edge business model by (1) transforming the role, skills, and style of the digital leader, (2) realizing a digital organization, including governance, vision, values, structure, culture, and decision processes, and (3) adjusting people management, virtual teams, knowledge, and communication and collaboration on the individual level.”
de Waal et al. (2016)	Information Systems research	Conference paper	Theoretical	A combination of transformational leadership style and the use of digital technology
Fisk (2002)	Management research	Research paper	Theoretical	A new leadership style that combines traditional with transformational leadership behaviours to generate organisational value in a digital world that is complex, fast-paced, connected, non-linear, virtual, and technology-enabled.
Mihardjo et al. (2019)	Management research	Research paper	Empirical	A combination of a leader’s digital mindset and digital competence to drive organizational transformation (in terms of business model innovation) supported by digital technology
Oberer and Erkollar (2018)	Management research	Research paper	Theoretical	“Digital leadership (leadership 4.0) is a fast, cross-hierarchical, team-oriented, and cooperative approach, with a strong focus on innovation. The personal competence of the leader, their mindset as well as their ability to apply new methods and instruments such as design thinking, are critical dimensions for 4.0 leaders.”
Klein (2020)	Management research	Research paper	Theoretical	“Digital leadership means leading the digital transformation process but also leading an organization in a digital environment depending on which digital maturity level the organization has.”
Sagbas and Erdogan (2022)	Management research	Research paper	Theoretical	“Digital Leadership is a leadership style that focuses on implementing digital transformation within an organization. It enables enterprises to digitize their work environments and learning cultures.”
Wasono and Furinto (2018)	Management research	Research paper	Empirical	“In terms of the digital leadership, the concept is created by combining the leadership skill and the digital capability to optimize the benefit of digital technology in order to increase the business performance.”
Zhong (2017)	Educational research	Research paper	Empirical	“…using instructional technology, including digital device, service, and resources, to inspire and lead school digital transformation, create and sustain digital learning culture, support and enhance technology-based professional development, provide and maintain digital organization management, and facilitate and manage digital citizenship.”
Sheninger (2014)	Educational research	Book chapter	Theoretical	“Digital leadership can thus be defined as establishing direction, influencing others, and initiating sustainable change through the access of information, and establishing relationships in order to anticipate changes pivotal to school success in the future. It requires a dynamic combination of mindset, behaviours, and skills that are employed to change and/or enhance school culture through the assistance of technology.”

As Table 1 depicts, definitions of DL stem from dispersed research areas such as information systems (IS), management and educational research. Most definitions from IS and management research mention the goal of a successful digital transformation by focusing on adjusted or new mindsets, competencies, skills, and behaviours of leaders. Interestingly, the oldest description of DL (Fisk, 2002), which is related to the Upper Echelons Theory (Hambrick and Mason, 1984; Erhan et al., 2022), developed independently of e-leadership (Avolio et al., 2000, 2014) pointing to their conceptual segregation. Further, only two definitions (El Sawy et al., 2016; Eberl and Drews, 2021) explicitly take different organisational levels into consideration to achieve strategic change in DT contexts. We conceptualise DL based on Eberl and Drews (2021) as we appreciate the multi-level character of their definition which covers the personal, individual (leader-follower interactions) and organisational level of DL. Besides, we value the differentiating character of this definition from e-leadership defined as *“a social influence process mediated by advanced information technologies (AIT) to produce a change in attitudes, feelings, thinking, behavior and/or performance of individuals, groups, and/or organizations”* (Avolio et al., 2014, p: 107). From a historical context, the definition of e-leadership addresses a different maturity state of digital technologies (strategic computing) in which communication technology was used to enhance the effectiveness of individuals and distributed groups, especially by linking enterprise systems with the then emerging Internet. This entails the conceptual perspective of technology as an emergent force which focuses on dynamic interactions between individuals or organisations and technology over time (Cascio and Montealegre, 2016). E-leadership therefore happens in a context in which work is mediated by information technology, especially in terms of communication and the collection and distribution of information which alters power relationships between leaders and followers due to the rising transparency and interconnectedness, and which requires new leadership behaviours to sustain trustful relationships (Avolio and Kahai, 2003). In contrary, the definition of DL is embedded into the age of ubiquitous computing (Schwarzmüller et al., 2018; Kane et al., 2019; Petry, 2019; Klus and Müller, 2021) implicating that digital technologies are pervasive and merge the physical with the digital world which leads to blurring boundaries such as between nations, organisations, customers, and partners. This entanglement perspective goes beyond the emergent force view of technology and describes that technology is intrinsic to everyday social interactions (Cascio and Montealegre, 2016). For the definition of DL, this broadens the scope meaning that the context of DT not only changes how business is executed by digital technologies but moreover how DT alters the fundamentals of organisations in the sense of their business model, structures, processes, and culture (Cortellazzo et al., 2019; Eberl and Drews, 2021).

Next to terminological considerations, organisational capabilities associated with DL represent another dimension for conceptualisation. Compared to the traditional understanding of leadership (Fisk, 2002; Erhan et al., 2022), the context of DL typically requires leaders to be equipped with new leadership capabilities for the strategic success of organisations (Kane et al., 2019; Franco, 2020; Klus and Müller, 2021) which is well accentuated by Erhan et al. (2022): *“However, the role of leadership requires new capabilities to obtain a secure sustainability for the organizations, as the technological progress introduces many changes to the organizations, such as digitalization of work and the workplace.”* However, this does not imply a complete abandonment of traditional leadership capabilities. To effectively guide organisations into a digital business world, digital leaders must combine traditional and new leadership capabilities (Kane et al., 2019).

For the conceptualisation of DL-related capabilities, we follow Benitez et al. (2022) who base DL capabilities on the RBV, and who consider DL capabilities as lower-order capabilities which indirectly impact firm performance through higher-order organisational capabilities (e.g., platform digitisation capability). This capability perspective underlines the micro-foundational approach of DL-related capabilities pursued in this study. Related studies emphasise the role of managers as drivers behind dynamic capabilities and analyse the managerial impact on strategic change (Adner and Helfat, 2003; Kor and Mesko, 2013; Helfat and Martin, 2015; Teece, 2016). As an example, Helfat and Peteraf (2015) as well as Hodgkinson and Healey (2011) show how a leader’s cognitive and psychological capabilities serve as underpinnings of dynamic capabilities.

Literature on DL capabilities majorly covers micro levels (individual, dyadic, teams) which goes in line with current research reflections of the field (Cortellazzo et al., 2019; Eberl and Drews, 2021). Due to terminological fuzziness (e.g., creativity declared as a leadership competence; Schwarzmüller et al., 2018; Kane et al., 2019; Petry, 2019; Klus and Müller, 2021) and as a trait (Schwarzmüller et al., 2018; Kane et al., 2019; Petry, 2019; Klus and Müller, 2021), we clustered DL capabilities identified in literature into characteristics, skills, behaviours and roles (coded leadership behaviours), and functions (responsibilities) of digital leaders (Table 2).

**Table 2:** Overview of DL capabilities.

Author/s & type	Leadership objective	Characteristics	Skills	Behaviours / roles	Functions
Benitez et al. (2022) Research paper (empirical)	Increasing organisational innovation performance by digitalising a firm’s platform facilitated by developing DL capabilities	Visionary / forward-looking Enthusiastic Integrative. Ambidextrous	Practical, hands-on digital skills (to lead DT programs and to act as an IT-business translator). Market, business, and strategic leadership skills	Selling and aligning on a common vision. Leading digital integration processes related to IT infrastructure, business process, data	Choosing and keeping the right team. Performing structural changes. Translating between IT and business contexts
Kane et al. (2019) Research paper (empirical)	Strategically prepare firms for DT-related organisational change by blending traditional and new leadership capabilities	Forward-looking Adaptable Change-oriented Open-minded Innovative	Strategic thinking: anticipate and evaluate trends, develop strategy General digital literacy (to assess business value of technology) Communication: articulate value of change	Providing a transformative vision and purpose Empowering people to think and act differently Getting people to collaborate across boundaries	Taking ownership for DT initiatives Attracting and developing talent Creating conditions and culture to experiment Creating a culture of distributed leadership and experimentation
Fisk (2002) Research paper (theoretical)	Leading organisations in complex, dynamic environments with a new leadership approach	Open to new possibilities Constantly curious Visionary Engaging / coaching Fusing / network-oriented Collaborating	Complexity management skills (holding different perspectives simultaneously, making sense of complex business contexts to create value for the organisation) Digital capacity (“techno-savants”: deep understanding of technology and its impact on markets and the organisation)	Formulating and empowering employees towards a strategic vision; giving purpose Transformational leadership behaviour (contextually combining traditional leadership with transformational perspective; actively seeking for self-transformation to transform the organisation; heavily investing resources for radical changes; embracing failures as opportunities for learning and reinvention)	Constantly orchestrating redesign of internal processes, business model and organisational structure Balancing order and disorder such as short-term business delivery vs. long-term digital-related business transformation Seeking collaborations and partnerships (internal / external)
Weber et al. (2019), Weber et al. (2022) Research paper (empirical)	Mastering DT-related organisational change (taking employees along the DT journey) by showing complementary leadership roles and behaviours	–	–	Behavioural complexity of leaders to take different, partly competing roles to achieve optimal change DT-related outcomes Task-oriented roles: digital pioneer, innovator, manager People-oriented roles: enabler, mentor, pioneer, digital mentee, networker	–
Klus and Müller (2021) Research paper (empirical)	Successfully cope with DT-related challenges by showing certain leadership characteristics and skills	Flexible Committed Creative	Think and act entrepreneurially (Self-)organisation skills IT skills Ability to motivate others Ability to decelerate	–	–
Petry (2019) Book chapter (theoretical)	Successfully guide firms in the digital economy by adapting leadership on organisational and lower-organisational levels	Networked Open Participative Agile Trusting	Strategic thinking: evaluate digital trends, develop strategy Business skills: e.g., innovation management, agile methods Digital skills: digital media literacy, data analytics (for data-based decisions)	Ambidextrous leadership behaviour (e.g., transformational, transactional, servant, or democratic style) Formulating a digital vision and digital strategy	Redesigning structures / processes Developing future skills and an agile culture Leading transformation programs
Westerman and McAfee (2012) Research paper (empirical)	Mastering organisational challenges of DT by building digital and transformation management capabilities	–	–	Communicating a transformative vision Driving employee engagement for a shared vision	Designing a digital governance model to lead the DT journey Shaping IT-Business relationships
Buvat et al. (2018) Research paper (empirical)	Mastering organisational challenges of DT by building digital and leadership capabilities	–	–	Promoting vision and purpose Enabling employees	Setting up governance structures Establishing a digital culture and employee engagement Shaping IT-Business relationships
Bennis (2013) Research paper (theoretical)	Instrumenting digital-driven transparency to react upon environmental dynamics, especially by the adaptive capacity of a leader	Adaptive capacity: resilient, feedback-oriented, open towards the new and learning from failures	–	–	–
Schwarzmüller et al. (2018) Research Paper (empirical)	Strategically prepare firms for DT by adjusting to changes of work design and leadership	Resilient Learning-oriented Result-oriented Participative Creative	Complexity management skills Creativity and problem-solving skills Communication skills IT skills Intercultural and language skills Remote leadership skills	Acting as a role model for employee health Motivating and inspiring employees Participative leadership behaviour (increased autonomy for and trust in employees) Relationship-oriented leadership behaviour: coaching and enabling; individualized employee consideration; increased networking and teambuilding	Satisfying employee flexibility requirements Fostering self-organized team setups Fostering internal and external collaboration Promoting and directing change Promoting agility and life-long learning Developing talent
Gilli et al. (2022) Research Paper (empirical)	Effectively manage DT by harnessing the chances of digital technologies for the organisation	Proactive Creative	Cognitive skills: communication, problem solving, analytical skills etc. Interpersonal skills: collaboration, team leadership, relationship management Business skills: project management, technological expertise, Big data analysis Strategic skills: strategic thinking, customer orientation, change management etc.	–	–

Fisk (2002) initially described the novel leadership context with a business environment that is complex, ambiguous, fast-paced, connected, non-linear, virtual, and technology-enabled leading to a rising attrition rate among business leaders. This context requires leaders to *“be able to hold several different perspectives at one time without being swamped by complexity.”* To cope with the new environment, Fisk (2002) advocates a new leadership style, called “digital leadership,” whose characteristics and behaviours go in line with the novel environmental qualities. In this sense, “digital” is not only interpreted as a technological phenomenon but also as a shift in mindset to create business value in new digital-related ways.

On a characteristics level, this requires digital leaders to be open to new possibilities, constantly curious, visionary, engaging, coaching, collaborating and network-oriented (Fisk, 2002). Referring to other research contributions, few overlaps exist such as being open-minded (Bennis, 2013; Kane et al., 2019; Petry, 2019), networked (Petry, 2019), participative (Schwarzmüller et al., 2018; Petry, 2019) and visionary (Kane et al., 2019; Benitez et al., 2022). The adaptive capacity is seen as distinctive for digital leaders and stands for several attributes such as being resilient, learning- and feedback-oriented, and open (Bennis, 2013), while similar attributes (adaptable, flexible, agile) are described in other studies (Schwarzmüller et al., 2018; Kane et al., 2019; Petry, 2019; Klus and Müller, 2021). To create business value in complex digital environments, several researchers mention innovativeness and creativeness as characteristical for digital leaders (Schwarzmüller et al., 2018; Kane et al., 2019; Klus and Müller, 2021; Gilli et al., 2022).

From a skill perspective, digital leaders need to blend traditional (e.g., communication and change management) and modern leadership skills relevant in DT contexts which are characterised by an increased pace of business execution, cultural shifts and tensions, workplace flexibilization, and greater expectations of productivity. For digital leaders, this especially demands strategic skills to assess and respond to technology-driven business trends with a suitable strategy and to direct the organisation towards it with a transformative vision (Kane et al., 2019). The importance of strategic and entrepreneurial skills is also highlighted by other researchers (Schwarzmüller et al., 2018; Klus and Müller, 2021; Gilli et al., 2022). To drive value creation and manage DT-related risks, leaders need to combine strategic and digital skills (Kane et al., 2019), however, researchers diverge considering the breadth and depth of digital skills. While Kane et al. (2019, p: 36) mention a high-level technological understanding in the sense of a *“general digital literacy, as opposed to hard-core technical skills,”* Fisk (2002) considers digital leaders as *“techno-savant leaders […] with a deep understanding of technology and its market impact.”* Other researchers such as Benitez et al. (2022) or Gilli et al. (2022) specify digital skills as competences to leverage technologies such as big data analytics, cloud computing, mobile app and web development, social media, ERP and CRM systems, and IT security. Finally, Klus and Müller (2021) speak of IT-skills unspecifically and highlight their positive impact to cope with digitalisation-related challenges such as personnel development, data privacy, and IT aversion of employees. Overall, researchers admit that skill requirements for leaders in DT contexts are increasing (Schwarzmüller et al., 2018) leading to a broad portfolio of digital, market, business, and strategic leadership skills (Benitez et al., 2022). On an intrapersonal level, researchers often point to digital leaders’ cognitive skills to solve business problems and to manage complexity and uncertainty (Fisk, 2002; Schwarzmüller et al., 2018; Gilli et al., 2022), especially driven by the digitalisation of work and changes in workplace communication and collaboration. As the importance of relationship-oriented leadership increases, *inter alia* due to the globalisation and flexibilisation of work, interpersonal skills (e.g., remote leadership skills) and intercultural skills also gather relevance for digital leaders (Schwarzmüller et al., 2018).

The elevated relationship orientation further reflects in digital leaders’ behaviours and roles. From this perspective, digital leadership requires a high degree of flexibility in the sense of contextually combining traditional (e.g., transactional leadership) and modern leadership behaviours (Fisk, 2002; Petry, 2019; Weber et al., 2022), however, with no consensus in literature regarding behavioural specifics. While Fisk (2002) describes the blending of traditional and transformational behaviours, Weber et al. (2022) or Weber et al. (2019) code leadership behaviours based on the Competing Values Framework (Quinn and Rohrbaugh, 1983) into task-oriented (e.g., digital pioneer) and people-oriented roles (e.g., mentor), and advocate for behavioural complexity of leaders to take different, partly competing roles to achieve optimal change outcomes in DT contexts. Further, Schwarzmüller et al. (2018) elaborate on key themes of DT-related change for work design and leadership. With respect to the leadership dimension, the authors favour a participative leadership behaviour in DT contexts by incorporating employee input in decision-making and distributing leadership tasks among employees fostering higher levels of autonomy. This ties to relationship-oriented leadership behaviour the authors identified as a macro-level change in DT leadership wherein leaders act as coaches and enablers, individually consider employee needs, and increasingly invest in networking and teambuilding. Finally, Petry (2019) mentions several leadership behaviours in the DL context such as transactional, transformational, servant, and democratic leadership and relates them unspecifically, i.e., without a differentiated consideration of leadership behaviours, to ambidextrous leadership. To achieve positive outcomes from an organisation’s innovation process, this concept denotes the flexible switching between complementary leadership behaviours towards individuals and teams, namely opening and closing behaviours which are either focused on exploration (innovation) or exploitation (efficiency; Rosing et al., 2011). Petry (2019) regards ambidextrous leadership as central to DL as it represents an effective mean to cope with rising leadership complexities in DT contexts, to balance short-term business delivery and long-term digital transformation (Fisk, 2002), and to align the business and IT world in organisations (Westerman and McAfee, 2012; Buvat et al., 2018; Benitez et al., 2022). Despite the academic dissent towards leadership behaviours, several commonalities exist which include the provision and alignment of employees towards a strategic vision (Fisk, 2002; Westerman and McAfee, 2012; Buvat et al., 2018; Kane et al., 2019; Benitez et al., 2022), enabling and empowering employees to think and act differently (Fisk, 2002; Westerman and McAfee, 2012; Buvat et al., 2018; Kane et al., 2019), and to collaborate across organisational boundaries (Kane et al., 2019). This is achieved by leadership functions such as developing talent, embracing an agile culture (Fisk, 2002; Buvat et al., 2018; Schwarzmüller et al., 2018; Kane et al., 2019; Petry, 2019), and driving organisational change as such by leading DT initiatives and programs, and by constantly orchestrating the redesign of organisational processes, internal and external collaboration structures and the business model (Fisk, 2002; Westerman and McAfee, 2012; Kane et al., 2019; Petry, 2019; Benitez et al., 2022). In summary, we characterise DL as an umbrella term which owns multiple, interlinked perspectives, and which represents a combination of various concepts of established leadership theories such as strategic leadership (Samimi et al., 2022), servant leadership (Sendjaya et al., 2008), transactional leadership (Antonakis and Day, 2017), transformational leadership (Bass and Avolio, 1993), authentic leadership (Gardner et al., 2011), and participative leadership (Huang et al., 2010; Zhu et al., 2018). This is consistent with results from Sow and Aborbie (2018) revealing that digital leaders should adopt a combination of leadership approaches to positively influence strategic outcomes of DT processes.

### 2.2. Service innovation and the dynamic capabilities view

This study defines service innovation as “*a new service experience or service solution that consists of one or several of the following dimensions: new service concept, new customer interaction, new value system/business partners, new revenue model, new organizational or technological service delivery system.*” (den Hertog et al., 2010, p: 494). Although an academic dissent about the constituents of service innovation such as the degree of change (incremental vs. radical) exists (Goduscheit and Faullant, 2018), the phenomenon increasingly gathers relevance in DT contexts (Vial, 2019). This means that research on service innovations acknowledges the significance and game-changing character of DT for organisations (Barrett et al., 2015; Lusch and Nambisan, 2015; Goduscheit and Faullant, 2018). Hence, to effectively realise service innovations, organisations need to exploit digital technologies and integrate them into their structures, processes as well as working and business models (Vial, 2019).

Referring to research perspectives on service innovations, we adhere to the synthesis approach which encompasses innovations both in service and manufacturing organisations, and which takes an integrative view not constrained to technological innovations (Witell et al., 2016). In this vein, service innovations can also trigger a competitive advantage for manufacturing organizations by making extensive usage of services but also by supplementing offerings with product-related services (Shelton, 2009). For the strategic management of service innovations, we follow the DCV which aims to explain differences in firms’ competitiveness under conditions of unpredictable change by purposefully changing their resource base (Teece et al., 1997; Eisenhardt and Martin, 2000; Helfat et al., 2009). The DCV is grounded in the Resource-based View (RBV), an established theory within management literature which proposes that firms should optimally exploit their own resources to enhance business performance (Barney, 2001). The RBV conceptualises resources to be “*heterogeneously distributed across firms*” and further assumes that these “*resource differences persist over time*” (Eisenhardt and Martin, 2000). A competitive advantage results from resources meeting the VRIN criteria (valuable, rare, inimitable, and non-substitutable; Eisenhardt and Martin, 2000). The DCV, compared to its initial version of the RBV, takes a more dynamic perspective by claiming that a set of VRIN-related resources is not sufficient for competitive advantage in rapidly changing business contexts. Instead, dynamic capabilities defined as “*the firm’s ability to integrate, build and reconfigure internal and external competencies to address rapidly changing environments*” (Teece et al., 1997, p: 518) are perceived as a cornerstone to gain a competitive edge. Teece (2007) further extended the DCV by a microfoundations perspective which aims to reveal lower-level phenomena of dynamic capabilities such as in terms of firm-specific skills as well as organisational processes and structures that make up the foundation for a firm’s competitive advantage. Teece (2007) states that dynamic capabilities can be disaggregated into a process consisting of (1) sensing business opportunities and threats, (2) seizing opportunities, and (3) transforming or reconfiguring a firm’s resource base. In short, dynamic capabilities can be described as sensing, seizing, and transforming capabilities which are built upon microfoundations to achieve a sustained competitive edge (Khan et al., 2020a,b).

Within the service innovation context, the DCV perspective is suitable as service innovations provide an intangible and firm-specific character matching to the characteristics of the DCV (den Hertog et al., 2010). Here, den Hertog et al. (2010) applied the DCV to conceptualise dynamic capabilities that organisations from a service and manufacturing background must possess to repeatedly create and market service innovations, so called dynamic service innovation capabilities (DSICs). This type of dynamic capabilities, which contains the trilogy of sensing, seizing, and transforming, allows organisations to quickly adapt to changing environments fostering their sustainable competitive advantage. In this context, the authors describe six DSICs for realising service innovations: signalling user needs and technological options, conceptualising, (un-)building, (co-)producing and orchestrating, scaling and stretching, as well as learning and adapting. The DSIC of signalling describes the ability to anticipate user needs and to signal new technological options to innovate a firm’s service portfolio. Conceptualising refers to an organisational ability to transform a new service idea into a viable service offering which includes aspects such as the target audience and service pricing. (Un-)bundling represents the ability to configure existing service elements into a new service offering by bundling or unbundling services. Co-producing and orchestrating defines a DSIC to manage service innovations across organisational boundaries and to engage in service alliances or networks. Scaling and stretching constitutes the ability to diffuse a newly created service firm- and market-wide by using marketing measures. Learning and adapting finally express a firm’s ability to deliberately reflect about how service innovations are currently managed and to derive adaptive measures (den Hertog et al., 2010).

In context of our research, we aim to shed light on the intersection of DL and service innovation. Related research refers to Benitez et al. (2022) who show the positive impact of an organisation’s DL capability on innovation performance by digitalising a firm’s platform. Further research has shown leadership’s positive influence on organisational innovation, especially with regard to leadership behaviours (Rosing et al., 2011; Jada et al., 2019; Afsar and Umrani, 2020) and skills (Schoemaker et al., 2018; Kane et al., 2019; Meissner and Shmatko, 2019; Ahmed and Harrison, 2021). From a service innovation perspective, relevant studies address the mediating role of leadership between service innovations and service firm competitiveness (Anning-Dorson, 2016), the positive impact of servant and transformational leadership on service innovation behaviour (Michaelis et al., 2010; Kao et al., 2015; Karatepe et al., 2019), and the positive impact of leadership behaviour on service innovation implementation (Chiu, 2018; Urquhart et al., 2018).

**Figure 1:**
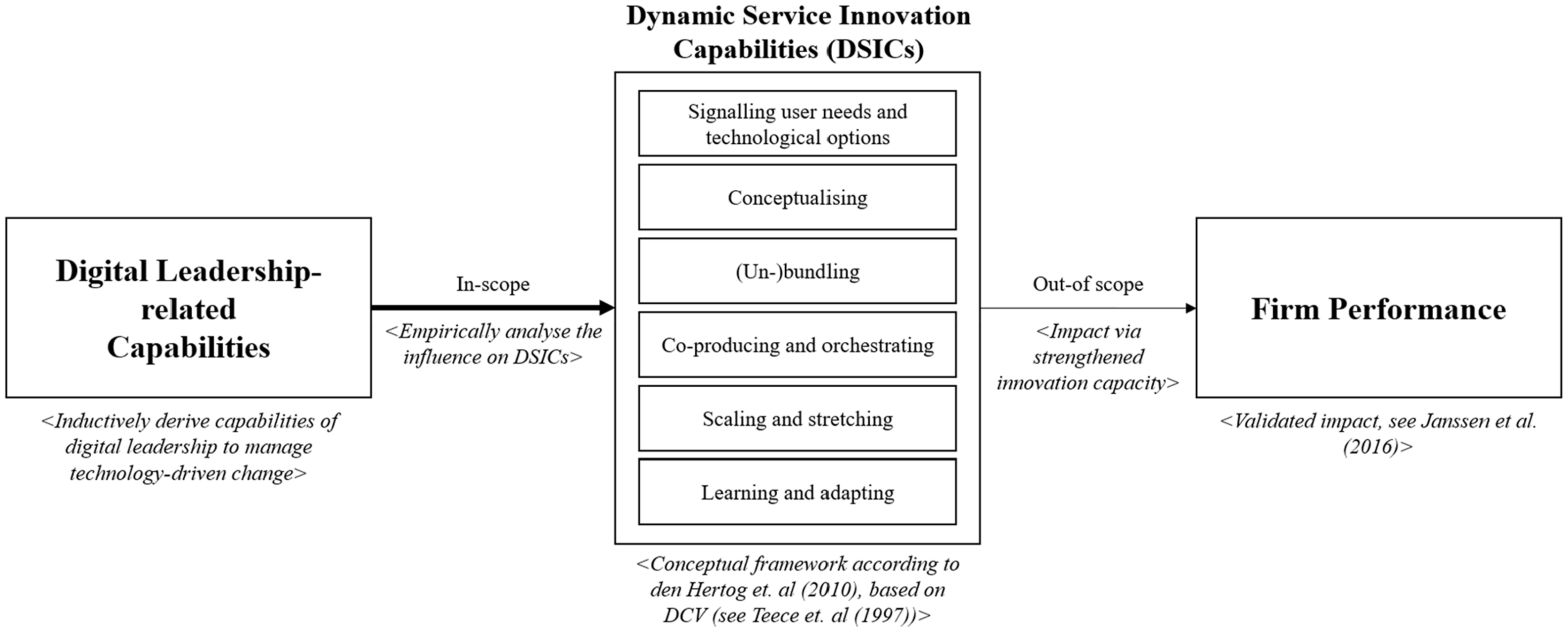
Research framework.

Given the positive influence of leadership on organisational (Rosing et al., 2011; Jada et al., 2019; Afsar and Umrani, 2020) and especially on service innovation (Anning-Dorson, 2016), we aim to draw a link between DL-related capabilities and DSICs. This link is conceptually depicted in Figure 1 which represents our research framework. Following this structure, we inductively investigate DL-related capabilities and empirically connect them to the six DSICs. This link delivers insights about the extent to which certain DL-related capabilities serve as a determinant for a strengthened service innovation capacity. As DSICs are considered as an effective measure to achieve qualitative and repeated service innovations in fast changing business environments (den Hertog et al., 2010), we consider them as an indicator to effectively manage change in DT contexts. Although DSICs own a positive impact on firm performance (Janssen et al., 2016), we do not include an outcome perspective of DL-related capabilities in the scope of our study.

## 3. Materials and methods

This study aims to scrutinise how DL-related capabilities influence the management of technology-driven change by leveraging service innovations. For this objective, we deem a qualitative research approach as suitable. Following Corbin and Strauss (1990), we focus on building theory and in this relation, Bluhm et al. (2011) further point out that a qualitative research approach can be applied to not only gather understanding of organizational processes but also to collect experiences from interviewees. Our data analysis includes a Grounded Theory approach (Corbin and Strauss, 1990), more specifically the Gioia methodology (Gioia et al., 2013). In this regard, a key aspect is to inductively derive concepts to build on existing theory or to establish new theory (Suddaby, 2006; Gioia et al., 2013). The basis for the analysis consists of 39 semi-structured expert interviews with top- and mid-level managers from Germany representing a managerial perspective to ensure a comparative basis regarding legal and political parameters. The interviewees correspond to SMEs and large firms of different industries which we reason with the DT context requiring innovative procedures of all firms, independent of size and industry. Regarding data analysis, 27 semi-structured interviews firstly delivered a data structure representing a taxonomy of DL-related capabilities in terms of a digital leader’s personal, social, and organisational capital. In a second step, 12 out of the 27 experts were interviewed again contributing to a conceptual framework which illustrates the influence of DL-related capabilities on DSICs. This multi-step data collection and analysis procedure based on a broad sampling strategy resembles the research methodology conducted by Warner and Wäger (2019).

### 3.1. Data sampling

The basis for this study consists of a purposefully derived sample of German service and manufacturing organisations of different sizes. Following Cresswell and Plano Clark (2011), experts were identified and selected based on their knowledge and experience in leading service innovation projects in the course of digitally transforming their organisation and partners. According to the Federal Ministry for Economic Affairs and Climate Action (2022), the offering of services within the core business of organisations currently accounts for creating 75% of jobs and constitute for 69% of Germany’s gross domestic product generating attractiveness for service-related studies. The close interlink of services within the industrial value creation is deemed a relevant success factor for Germany as a traditionally industry-oriented nation to maintain its competitiveness and innovativeness, *inter alia* by offering service-infused product bundles or collaborating with service organisations (Federal Ministry for Economic Affairs and Climate Action, 2022). Therefore, this study concentrates on both service and manufacturing organisations in Germany differentiating in company sizes such as SMEs and large companies. Using the definition of the European Commission, SMEs employ less than 250 people and at the same time own an annual turnover of less than EUR 50 million and/or an annual balance sheet total not surpassing EUR 43 million (European Commission, 2003). In general, only service and manufacturing organisations were chosen which operationalise service innovation projects to drive technology-driven change based on the above-mentioned definition of den Hertog et al. (2010). For this purpose, up-front meetings with representatives were held to ensure a common understanding of the phenomena of interest. In the present study, SMEs and large service and manufacturing firms are equally distributed. By applying selection criteria related to service innovation projects and firm size, this study takes an ample perspective when it comes to the different industries considered. This is due to Germany’s economy which owns cross-industry characteristics accommodating both service organisations with classic service offerings (e.g., advertisement and marketing or finance, insurance, and real estate) and manufacturing organisations with product-related service offerings (e.g., metal and electronics or traffic and logistics). For the classification of industries, we followed the Federal Statistical Office of Germany (2022) which includes German service firms such as advertisement and marketing, energy and environment, finance, insurance, and real estate, as well as manufacturing companies operating as metal, electronic, and vehicle manufacturers and the traffic and logistics sector. Following the organisational sampling, interview candidates from the identified service and manufacturing organisations were purposefully selected using a criterion-i sampling strategy. As stated by Palinkas et al. (2015), this sampling method is used to select experts meeting a predetermined criterion, and hence, possess knowledge and experience concerning the phenomenon of interest. For our study, the criterion of interest is experts possessing knowledge and experience in operationalising service innovation projects which allows them to provide detailed and generalizable information about the management of technology-driven change in organisations. To select knowledgeable interviewees with profound experience in this matter, we applied three criteria. First, all experts must be actively engaged in executive decision processes within their organisation. To involve not only a mere top-level strategic viewpoint, but also a tactical point of view of how DL is performed in organisations, mid-level managers were also included in the sample (Langley and Abdallah, 2015). Second, experts must provide either a three-year experience in being disciplinary managers, and/or a three-year experience in occupying lateral leadership roles such as SCRUM Master or Product Owner. Third, experts need to own a responsibility for digital organisational development for at least 3 years. This includes duties such as developing agile organisational designs, establishing digital cultures, and implementing digital leadership models. Applying the selection criteria resulted in 27 service and manufacturing organisations with 39 interview partners from top- and mid-level management.

For the first interview round, organisations were contacted from which we received 27 confirmations from service (*n* = 17) and manufacturing (*n* = 10) organisations. From the 27 organisations, 15 own a SME background whereas 12 are regarded as large companies. In the second interview round, these organisations were contacted again, and 12 experts accepted the invite. These experts belong to service (*n* = 8) and manufacturing (*n* = 4) organisations whereas six firms hold a SME background, and six entities relate to large companies. Interviewing first-round experts again ensures that understanding of the context is present so that the influences of DL-related capabilities on DSICs can be described precisely. Table 3 summarises information about the whole data set.

**Table 3:** Description of the data sample and set.

Data sample	
Country	Germany
Industry	Service and manufacturing companies
Sectors	Service:
	Advertisement and marketing; Economy and politics; Energy and environment; Finance, insurance, and real estate; Groceries and nutrition; Internet; Pharma and health; Trade; Society.
	Manufacturing:
	Metal and electronics; Technic and communication; Traffic and logistics
Interview partners	Top- and mid-level managers
Selection criteria	
Company industry	German Service firms due to their classic service offerings
	*First interview round (27 interviews): n = 17*
	*Second interview round (12 interviews): n = 8*
	German Manufacturing firms due to their growing product-related service offerings
	*First interview round (27 interviews): n = 10*
	*Second interview round (12 interviews): n = 4*
Company size	SMEs with 250 employees and EUR 50 million turnover and/or EUR 43 million balance sheet total
	*First interview round (27 interviews): n = 15*
	*Second interview round (12 interviews): n = 6*
	Large companies with 250 employees and EUR 50 million turnover and/or EUR 43 million balance sheet total
	*First interview round (27 interviews): n = 12*
	*Second interview round (12 interviews): n = 6*
Interviewees	Strategic top-management perspective
	*First interview round (27 interviews): n = 9*
	*Second interview round (12 interviews): n = 4*
	Tactical mid-management perspective
	*First interview round (27 interviews): n = 18*
	*Second interview round (12 interviews): n = 8*
Experience and responsibility	Experience (at least 3 years) in being disciplinary managers within their company
	Experience (at least 3 years) in lateral leading roles within their company
	Responsible for digital and organisational development within their company
Data set	
Total interviews	39 interviews in 27 organisations
	*First interview round: 27 interviews*
	*Second interview round: 12 interviews*
Total interview duration	33 Hours 28 Minutes 10 Seconds
Period of data collection	March 2021 – February 2022

### 3.2. Data collection

During data collection, the 39 interviews were conducted in German language over a period of 11 months between March 2021 and February 2022 lasting between 42 min and 71 min. The interviews in each round were based on a guideline which was regularly revised following Gioia et al. (2013). All interviews were recorded and transcribed (Table 4).

**Table 4:** Interviewees.

Company	Size	Industry (Sector)	Position (Level)	2nd round
1st interview round				
Company A	SME	Service (Internet)	Chief Executive Office (Top)	Yes
Company B	SME	Service (Advertisement and marketing)	Director R&D (Mid)	No
Company C	Large	Service (Energy and environment)	Vice President Learning and Innovation (Mid)	Yes
Company D	Large	Manufacturing (Traffic and logistics)	Global Head Digital Transformation (Mid)	Yes
Company E	Large	Manufacturing (Metal and electronics)	Manager R&D (Mid)	Yes
Company F	SME	Manufacturing (Technic and communication)	Director Innovation (Mid)	Yes
Company G	Large	Service (Trade)	Chief Executive Office (Top)	Yes
Company H	SME	Service (Internet)	Head of Digital Transformation (Mid)	No
Company I	SME	Service (Groceries and nutrition)	Chief Executive Office (Top)	Yes
Company J	Large	Service (Internet)	Executive Director Innovation and R&D (Mid)	No
Company K	SME	Service (Trade)	Project Manager Innovation (Mid)	No
Company L	SME	Service (Finance, insurance, and real estate)	Head of Digital Transformation (Mid)	No
Company M	Large	Manufacturing (Traffic and logistics)	Vice President Digital Transformation (Mid)	Yes
Company N	SME	Service (Finance, insurance, and real estate)	Project Manager Innovation (Mid)	No
Company O	SME	Manufacturing (Metal and electronics)	Head of Digital Development and Innovation (Mid)	No
Company P	Large	Service (Finance, insurance, and real estate)	Chief HR Officer (Mid)	No
Company Q	Large	Service (Pharma and health)	Chief Executive Office (Top)	No
Company R	Large	Manufacturing (Metal and electronics)	Chief Executive Office (Top)	Yes
Company S	Large	Service (Finance, insurance, and real estate)	Chief Executive Office (Top)	Yes
Company T	Large	Service (Finance, insurance, and real estate)	Senior Consultant Digital Transformation (Mid)	Yes
Company U	SME	Manufacturing (Metal and electronics)	Chief Executive Office (Top)	No
Company V	Large	Service (Groceries and nutrition)	Project Manager Innovation (Mid)	No
Company W	SME	Manufacturing (Traffic and logistics)	Chief Executive Office (Top)	No
Company X	SME	Service (Trade)	Chief Executive Office (Top)	Yes
Company Y	SME	Service (Finance, insurance, and real estate)	Principal Consultant IT (Mid)	No
Company Z	SME	Manufacturing (Traffic and logistics)	Project Manager R&D (Mid)	No
Company AA	SME	Manufacturing (Technic and communication)	Program Director R&D (Mid)	No

All names of companies and interviewees are anonymised throughout the study as confidentiality was guaranteed to all interview partners. In the first interview round, 27 experts were interviewed. Top-level managers (*n* = 9) worked in service SMEs (*n* = 3), large service organisation (*n* = 3), manufacturing SMEs (*n* = 2) and large manufacturing organisations (*n* = 1). Mid-level managers (*n* = 18) worked in service SMEs (*n* = 6), large service organisation (*n* = 5), manufacturing SMEs (*n* = 5) and large manufacturing organisations (*n* = 3). In the second interview round, 12 experts were interviewed. Top-level managers (*n* = 6) worked in service SMEs (*n* = 3), large service organisation (*n* = 2) and large manufacturing organisations (*n* = 1). Further, mid-level managers (*n* = 6), large service organisation (*n* = 2), manufacturing SMEs (*n* = 1) and large manufacturing organisations (*n* = 3).

The 27 semi-structured interviews from the first interview round were conducted with nine top-level and 18 mid-level managers who were actively engaged in and knowledgeable about service innovations projects. We interviewed top managers to obtain a holistic and strategic perspective of how organisational leaders encourage and facilitate DL and how they foster technology-driven change by operationalising service innovation projects. Interviews with mid-level managers were conducted to get a practical and tactical understanding of how organisations operationalise service innovation projects to reach technology-driven change within the firm. Superiors identified mid-level managers from different backgrounds (e.g., SCRUM masters or Product Owner) especially experienced in leading service innovations projects to drive DT in their field. Both hierarchical levels were regarded as experts for our research as their human capital is strongly linked to their knowledge and experience referred to service innovations, digital leadership and the management of DT (Helfat and Peteraf, 2015; Warner and Wäger, 2019). The incorporation of top- and mid-level managers in a service innovation context follows the view of Eisingerich et al. (2009) allowing to extract information concerning the communication, action, exploitation, and management of business opportunities *via* service innovation projects. In line with Gioia et al. (2013), a semi-structured interview guideline was used for the interviews to find answers to our research question and open questions were asked to not guide participants towards a specific direction. Among others, first-round interviewees were asked to explain how leadership changed against the backdrop of DT and COVID-19, to describe how they define DL, and to elaborate on how their organisation implements DL in current service innovation projects. Further, a focus was placed on the assessment of which capabilities are needed to be regarded as a digital leader. Organisations and experts were included in the first interview round until theoretical saturation was reached. Interview findings served as an input to develop the taxonomy of DL-related capabilities. The taxonomy provided a basis for a second round of interviews to answer the research question of how DL-related capabilities influence the management of technology-driven change by leveraging service innovations. In this second round, all initial 27 experts were contacted again resulting in 12 experts available for a second interview round. From the 12 positive responses, four represented top managers and eight owned a position in mid-level management. By using the definition from den Hertog et al. (2010), a common understanding of service innovation was created together with the experts at the beginning. All interviewees were then asked to assess which DL-related capabilities, based on the taxonomy, enable technology-driven change given the context of a service innovation project. From this point, the experts described their service innovation projects. As an example, a mid-level manager of an energy provider explained his perspective of the project working as a Product Owner where he was responsible to achieve an improved customer experience by creating a digital payment system. As another example, one top manager of an automotive company from the metal and electronics sector described the streamlining of several digital touchpoints of one of their services in their service innovation project. Besides the project reporting, experts were requested to elaborate on the influence of DL on the management of service innovations within their organisations, to describe processes to identify customer needs or technological trends at an early stage, to explain advantageous leadership behaviours for recognising service innovation potentials, and to depict on principles of collaboration relevant to conceptualise a service innovation. Moreover, interview questions also concerned how digital leaders can influence the development of a culture of continuous learning and the organisational adaptability in the context of service innovations.

### 3.3. Data analysis

Two researchers were actively engaged in the data analysis process which proceeded in two steps in correspondence to the two interview rounds.

**Figure 2:**
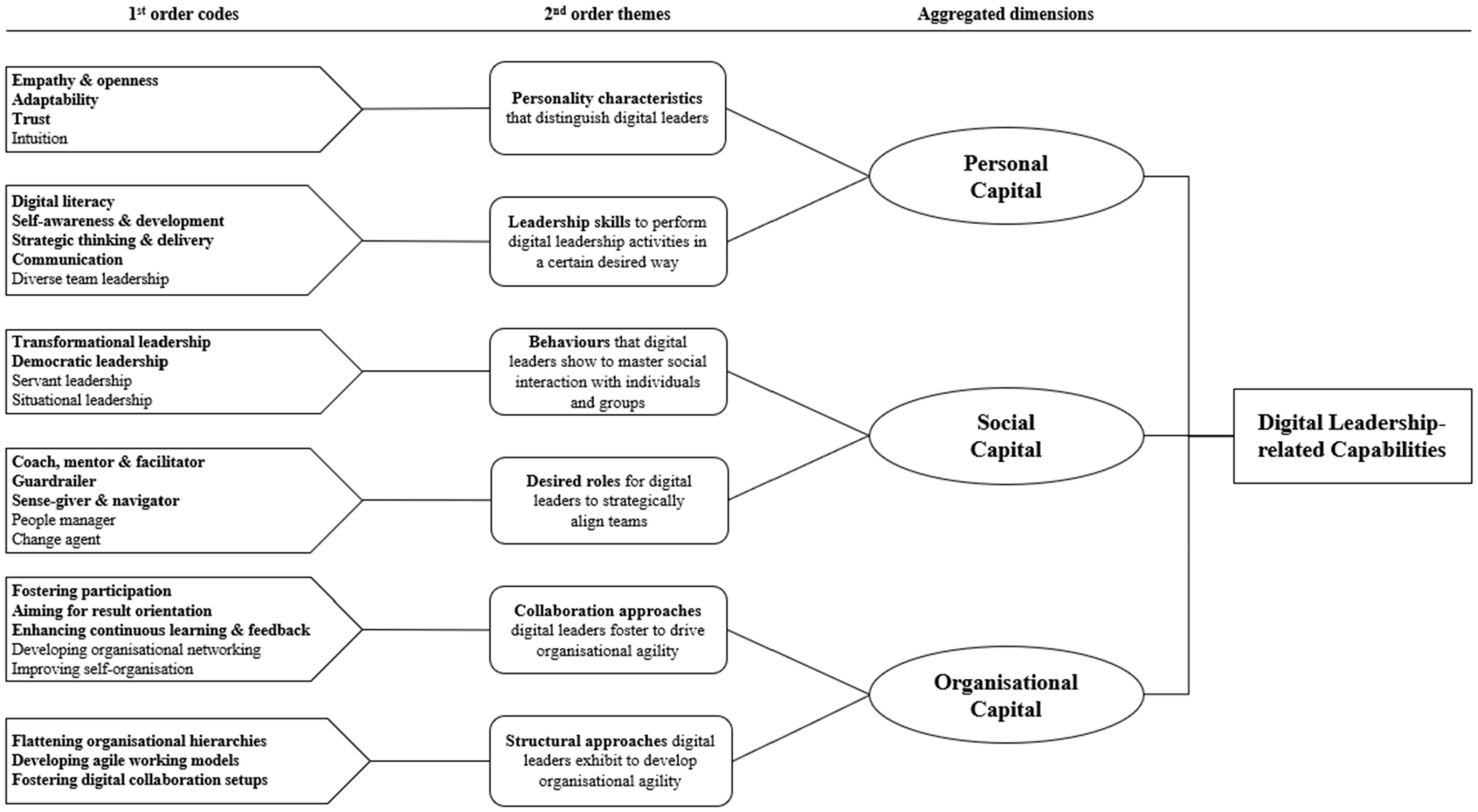
Taxonomy of DL-related capabilities. *Note:* Only first-order codes which were mentioned multiple times by experts and deemed relevant in managing DT operationalised by DSICs are marked in bold during the second interview round.

First, to analyse the 27 interviews, both researchers were involved in the coding process, whereas one researcher thematically kept distance and improved the outcome by challenging the analysis process such as by iteratively giving feedback on the coding system (Gioia et al., 2013; Crosina and Pratt, 2019). In this sense, one researcher acted as “devil’s advocate” to critically reflect upon the coding, asking for clarification and reconsidering newly developed themes within the data to improve the data structure (Crosina and Pratt, 2019). Following Corbin and Strauss (1990) and Gioia et al. (2013), the data analysis followed three steps presented in a linear but executed in an iterative way (Suddaby, 2006). In the first step, the transcribed interviews were analysed using open coding (Corbin and Strauss, 1990) with the help of the qualitative data analysis program MAXQDA. In this way, first order codes were created more descriptive in nature and adapted closely to the actual quotes. In the second step, axial coding was deployed to organise and group first order codes in a more theoretical manner (Corbin and Strauss, 1990; Gioia et al., 2013). Thus, through the ongoing revision process, first order codes were categorized in second order themes. For instance, the second order theme “Personality characteristics that distinguish digital leaders” was created by clustering the first order codes of “empathy & openness,” “adaptability,” “trust,” and “intuition.” In the third step, theoretical coding was applied to derive aggregated dimensions (Corbin and Strauss, 1990). Overall, some of the identified themes were more related to capabilities relating to the personal level, while others involved a social or an organisational perspective of a digital leader. Figure 2 presents the results of the data structure (taxonomy) of DL-related capabilities consisting of first order codes, second order themes and aggregated dimensions.

In the second step of the analysis, based on the findings presented in Figure 2, the 12 interviews from the second interview round were analysed by the same two researchers with the help of MAXQDA also following a three-step approach.

In the first step, experts were confronted with the derived taxonomy from the first interview round and asked to assess which first order codes are crucial to manage DT using an exemplary service innovation project from their organisation. The aim was to get an initial sense of which codes experts consider as relevant. Using open questions, interviewees were able to explain their answer and choose more than one first order code. In Figure 2, only codes mentioned more than two times considering the influence on DSICs are marked in bold. Hence, as an example for “behaviours that digital leaders show to interact with individuals and groups,” experts only regarded two first order codes (transformational and democratic leadership style) as crucial for managing DT operationalised by DSICs.

In the second step, to further accentuate experts’ opinion towards DL-related capabilities, interviewees were asked to explain the influence of the second order themes on DSICs. This step was performed as second order themes and DSICs resemble in their thematic granularity. In this vein, interviewees were invited to comment on the influence of every second order theme regarding the six DSICs. For instance, experts were asked to describe the influence of personality characteristic of a digital leader on the ability to understand and sense user needs, or to elaborate on the influence towards signalling technological options to innovate services. Experts answered these questions using first order codes to stress specific experiences or situations and related them to the second order themes.

**Figure 3:**
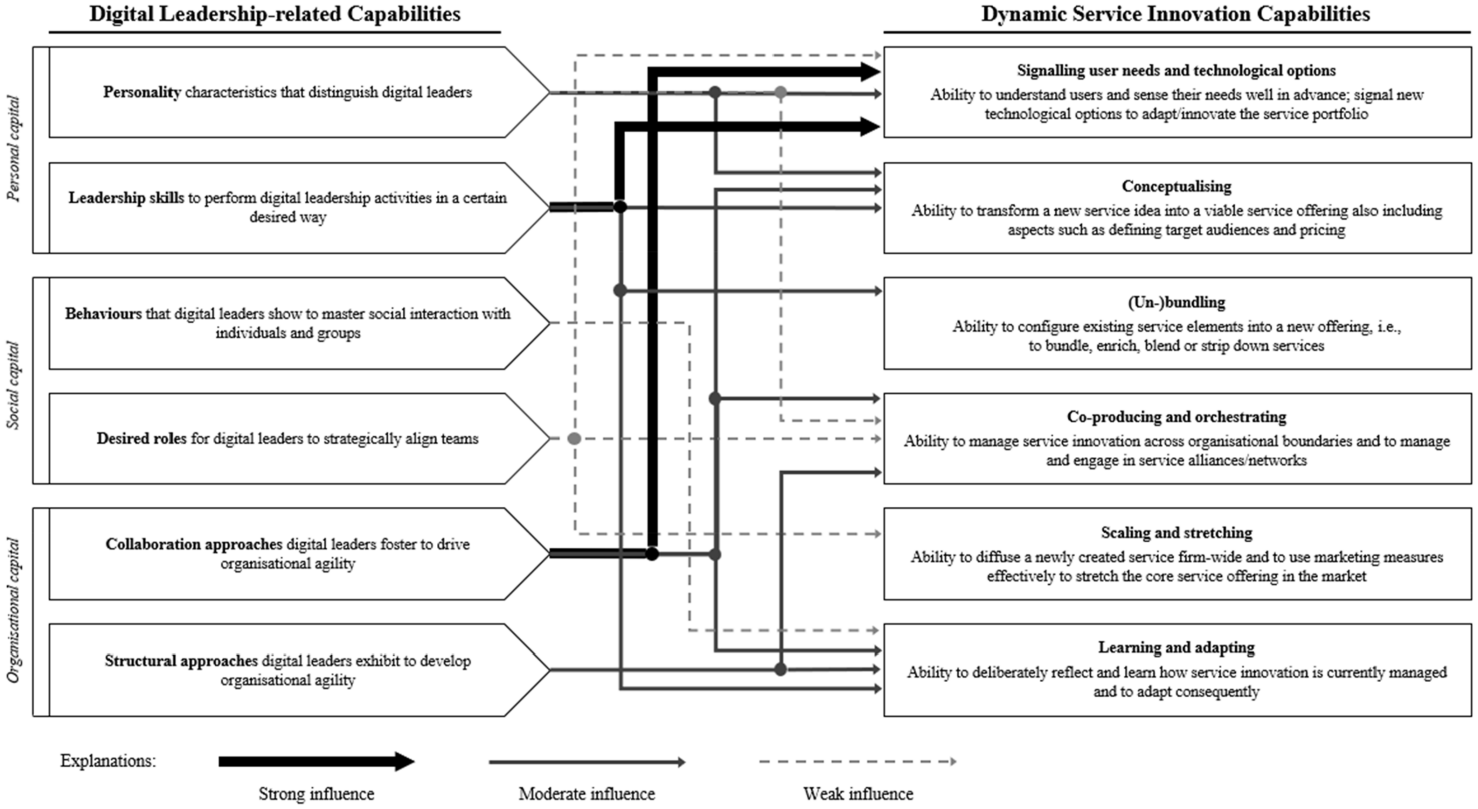
Influence of DL-related capabilities on dynamic service innovation capabilities. *Note:* All interviewees were exposed to the analysed DL-related capabilities of the data structure (taxonomy). Based on expert’s nominations, the thick lines represent a strong influence of digital leadership capabilities for technology-driven change on the DSICs. Thin lines represent a moderate influence, and respectively, dotted lines have a relatively weak influence on DSICs.

In the third step, nominations were analysed. Influences (arrows) were only counted whenever at least two experts explicitly described a second order theme in relation to one of the DSICs through first order codes. To ensure comparability of the influences, we mirrored all mentions of second order themes (in terms of first order codes) in the end and determined relative degrees of their influences on DSICs. For instance, referring to the personal and functional capability of a digital leader, we identified a strong and moderate influence on the DSIC of signalling user needs and technological options. This applies to personality characteristics with a moderate influence (*n* = 6 nominations) as well as leadership skills (*n* = 10 nominations) and collaboration approaches (*n* = 12 nominations) with a strong influence. Figure 3 illustrates the influence of DL-related capabilities in different degrees (strong, moderate, weak) on DSICs based on experts’ relatively coded nominations.

## 4. Results

The qualitative findings of this study are reflected by a taxonomy of DL-related capabilities relevant in DT contexts and a conceptual framework which illustrates their influence on the management of technology-driven change using the context of service innovation projects. Results show that DL-related capabilities are headed towards the personal, social, and organisational level and are differently evaluated by interviewees with respect to organisational setups shaped by DT. A digital leader’s personal capital addresses relevant characteristics and skills in DT contexts, whereas the social capital covers interpersonal aspects in terms of their behaviours and roles directed towards individuals and groups to achieve strategic organisational change. Finally, the organisational capital details structural und collaboration cultural approaches a digital leader pursues to develop organisational agility. According to the experts, the personal and organisational capital of a digital leader overall own a stronger influence on DSICs than the social capital.

### 4.1. Personal capital

With regards to the data structure, experts deemed the personality characteristics of empathy and openness, adaptability, and trust as crucial in a DT context. Referring to the influence on DSICs, we identify a moderate influence of personality characteristics on signalling user needs and technological options, as well as on conceptualising, and a weak influence on co-producing and orchestrating. Moreover, experts described digital leaders’ skills in terms of digital literacy, self-awareness and development, strategic thinking and delivery, and communication as relevant to manage DT-related change. Leadership skills own a strong influence on the DSIC of signalling and a moderate influence on conceptualising (un-)bundling as well as learning and adapting.

#### 4.1.1. Personality characteristics that distinguish digital leaders

Digital leaders express certain personality characteristics which influence DSICs, particularly the signalling of user needs and technological options, the conceptualisation, and the co-production and orchestration of a service innovation (den Hertog et al., 2010). According to the experts, **empathy and openness** were described as relevant for digital leaders. One top-manager explained that *“human closeness, for example, the interest in fellow co-workers and employees, is crucial to look beyond the professional context supporting team dynamics, team development processes and empathy for the whole organisational environment.”* Additionally, another expert suggested that digital leaders’ openness towards different ways of thinking and experimenting will ultimately lead to more sustainable success. Agreeing, one manager explained the following: *“Digital leaders must not only be open towards digital tools that employees bring to the workplace but also accept it. For instance, it is quite normal for someone who is digitally socialized to communicate with others via social media or communication software while being in a meeting. That was frowned upon in the old world, but the people who grew up like that can still listen and operate their cell phones in parallel.”* Empathy and openness are often seen in combination, especially considering the DSICs of signalling, as well as co-production and orchestration. Given the signalling capability, referring to the data, digital leaders break down their traditional perception about the management of service innovation processes. In other words, they show empathy towards customers and other stakeholders, and they must also be open to new ways of thinking to anticipate novel ideas and trends. Moreover, while anticipating customers’ concerns, empathic and open digital leaders can both understand the needs of the customer and develop an option to fulfil this need. Given co-production and orchestration, the experts mention that empathy and openness enhance internal collaboration with team members and external coordination with customers. In this regard, one top-level manager explicitly noted that, *“Empathy and openness represent the key to success in producing a successful service innovation outcome, developing and expanding digital ideas in a diverse team from idea to trial beyond the business case at the customer.”* Another DSIC-relevant characteristic is **adaptability**. One mid-level manager explained adaptability against the backdrop of DL: *“The number one pillar for a digital leader during the digital transformation is adaptability. He [digital leader] must be capable of change and needs to be able to deal with risks and be willing to experiment. This must be encouraged in all teams. However, a digital leader has to set the framework and promote it in the team. Overall, I believe that the need for adaptation has always existed. While working for this company quite some time I can say that we have actually always been changing, but the difference is that the cycles for change are becoming shorter and shorter due to digitalisation.”* While observing the data, digital leaders expose a certain level of adaptability to deal with internal and external uncertainties (e.g., the COVID-19 pandemic). Digital leaders base their decisions upon hypotheses and experiments in uncertain situations allowing them to adapt to changing business and regulatory environments. Given the co-production and orchestration of a service innovation, an adaptable digital leader can be advantageous. The experts noted that demonstrating adaptive attitudes towards novel ideas in context of service innovations across internal and external boundaries strengthens the ability to manage a successful outcome. According to the experts, this can be achieved by digital leaders who collaborate with teams and customers by agreeing on predefined structures and a respective mindset. In terms of **trust,** the interviewees acknowledged that trust represents an essential characteristic for digital leaders. In this sense, one top-manager described that *“in general, it is valuable to have trust. On the one hand, trust that you as a manager can achieve the target state, even if resistance arises at the beginning. And on the other hand, you need to have trust in your workforce that is to achieve the planned change. This also needs to be expressed to the employees at some point.”* Trust is evident in three DSICs including signalling, conceptualising, and co-producing and orchestrating. Regarding the signalling capability, customer needs can be detected, and a fitting solution can be created in a more efficient way due to a mutual trust relationship between customers and digital leaders leading to a transparent exchange of information. Further, trust plays an important role for conceptualising. This was described by the experts as constantly picking up both the team and the customer through a continuous trustful interaction between them to agree on a common target audience or pricing which leads transform a sole idea into a viable offering. Finally, in co-producing and orchestrating a service innovation, trust forms the foundation to engage in eye-to-eye communication with the team and customer, as well as to decide upon results and their quality. In this context, interdisciplinary work and the acceptance of other experts’ status are essential to optimise the planning and execution of the service innovation. In describing personal DL capabilities, experts also named **intuition**. With regards to intuition, many experts agreed on the fact that digital leaders need to listen to and rely on their intuition. One to-level manager noted that *“sometimes you just have a feeling about a digital change and then you have to persevere and follow up on it.”*

#### 4.1.2. Leadership skills to perform DL activities in a certain desired way

Based on the interview data, this capability especially influences the DSICs of signalling user needs and technological options, conceptualisation, (un-)bundling, and also adapting and learning (den Hertog et al., 2010). As shown by the data, **digital literacy** is crucial for digital leaders. One mid-level manager pointed out that digital leaders need to obtain a basic digital knowledge, such as working with digital tools for collaboration and workflow design, which he described with *“I believe that it is very important for leaders to deal with digital tools, processes and working methods, and perhaps also hardware.”* One top-level manager accentuated the strategic skills a digital leader must possess to assess the impact of digital technologies: *“Do I need to understand why a machine learning algorithm needs different hardware to work? No, the general manager does not have to. Maybe not even the digital department of the company, because it can make use of external specialists. But I do need to understand how this can change things in our lives, in our society and in our company…[otherwise] if a manager of an area does not deal with these topics or does not have an expert to deal with them, standstill of all business processes is inevitable.”* The digital literacy is important for signalling user needs and technological options during service innovation projects. In this vein, experts underlined that modern business contexts are characterised by a high volume of available data. For digital leaders, this requires skills related to digital technologies that are linked to both the capacity of digital leaders to raise the right questions towards data and to make data-driven insights accessible to stakeholders and customers. These insights support the sensing of user needs and the identification of the right technological option for the customer. Furthermore, the interviews demonstrated that digital leaders need **self-awareness and personal development**. In this way, one top-manager noted that *“in order to create self-awareness, our managers must collect feedback from their teams and take it with them to what they can do with it, hence, enable an open space where you receive open feedback and deal with it.”* In contrast, this was only partially confirmed by another top-manager: *“The concept of self-awareness is not enough for a digital leader. A turning point is personally developing and dealing with new technologies and software. A digital leader must be capable of being coached by an employee, endure an argument, or even switch roles. That did not exist in the past, however, this means I have to adjust to it because self-awareness and external awareness diverge.”* Especially the DSIC of learning and adapting is addressed by self-awareness and personal development. To learn and continuously adapt in service innovation contexts, digital leaders need to admit mistakes and to have the humility of not-knowing but trusting in the competences of the team and customers. Experts also mentioned **strategic thinking and delivery** as a relevant personal DL capability. In this vein, experts agreed that this capability is inevitable in times of increasing digitalisation, complexity, and uncertainty. Data show that a digital leader needs to take multiple perspectives to face these issues. One expert focused on this aspect: *“It needs a strategic mind behind linking IT, digital business initiatives and digital resources well with each other and deliver it,* e.g.*, to the team or customer. This will be the eternal challenge of using the bottleneck resources efficiently and not failing on the topic of digitalisation projects, so to say, to get even smaller initiatives through even though,* e.g.*, IT resources are allocated to large projects.”* Strategic thinking and delivery influences three DSICs. First, during signalling, the experts reported that digital leaders show the capacity of reasonably selecting from a multitude of information or data to generate customer insights. Based on these insights, a digital leader can frame a service innovation fitting to the customer concerns, as well as to the strategic orientation of the organisation. Second, relating to the conceptualisation capability, the experts noted that digital leaders collaboratively work out a strategic plan towards transforming a new idea into a service innovation. Defining how the initial service idea can be implemented from conceptualisation throughout the final offering is important in this context. Third, while unbundling or bundling, the interviewees explained that digital leaders need to (re-)configure existing service elements. Among others, this requires managing organisational obstacles leading to strategic decisions to either innovate within existing business structures or separately on a greenfield site. Furthermore, **communication** was mentioned as an essential DL capability. One top manager argued that communication is always a key capability for leaders in organisations and has nothing to do with digital transformation and its related challenges. However, this statement was opposed by another top-level manger: *“Especially in times of digitalisation and COVID-19, strong communication skills become a powerful tool for digital leaders to reflect themselves and effectively place messages to their employees that need to be conveyed. Moderation skills, goal-orientation as well as clarity in procedures become increasingly important in times where new digital tools or software are employed into business processes.”* Data shows that communication towards the team and customers is crucial for the DISCs of conceptualising, and learning and adapting. The experts mentioned that linked to the conceptualisation capability, communication at eye level, and the combination of different competences within the team and customer are key for the development of a viable service offering. Against the backdrop of learning and adapting, the interviewees demonstrated that continuous formats of reflection foster the incremental creation of a service innovation. In this regard, one expert explained: “*At the foundation [of a learning and adapting culture] digital leaders need to frame a transparent communication structure. The use of methodological retrospectives without looking for culprits is considered to be very important in the service innovation process.”* Experts further considered **diverse team leadership** as important, although no influence on DSICs could be identified in the data. In this context, one mid-level manager emphasised digital leaders’ excellence in remote leadership as *“we will end up in a hybrid working world within the service industry, so finding out what employees need, managing that and responding to it will become crucial, for instance, how can I lead mixed teams that have different attitudes and cultures in the future.”* For another top manager, the hybrid working setup was not going too far as the randomness of encounters with employees decreases in the digital world. In this sense, she argued that digital leaders must actively orchestrate physical touchpoints with their employees to achieve a high grade of productivity.

### 4.2. Social capital

The data structure illustrates that digital leaders should exhibit certain behaviours and roles. In this vein, interview data suggests that transformational and democratic leadership styles play a role in influencing DSICs, although this influence on the DSIC of learning and adapting is weak. In terms of desired roles of digital leaders, interviewees expressed the three roles of being a coach, mentor, and facilitator, being a guardrailer, and being a sense-giver and navigator as pertinent in DT contexts. However, DL roles turned out to only have a weak influence on the DSICs of signalling, co-producing, and orchestrating, and scaling and stretching.

#### 4.2.1. Behaviours that digital leaders show to master social interaction with individuals and groups

In the experts’ opinion, digital leaders’ behaviours are especially linked to the DSIC of learning and adapting (den Hertog et al., 2010). Almost every manager emphasized that traditional styles of leading employees and organizations are currently subject to a fundamental change. One top manager specified this: *“The cultural change we are facing concerning leadership means that we want to distance from a transactional leadership and move towards the direction of more servant, transformational, democratic and contextual styles of leading. And of course, that also requires that a certain mindset must be developed for this, a behaviour in which I then also develop a doing.”* In line with this behavioural switch, the experts highlighted that digital leaders should practise a **transformational leadership** described by one expert with: *“Today, we lead transformationally,* i.e.*, rather cooperatively, persuasively, no longer building up pressure on employees, but interacting and leading persuasively and empathically.”* The transformational leadership style in terms of role modelling towards employees especially relates to the capability of learning and adapting which is confirmed by one top-level manager: “*Again, this role modelling is extremely important, I think, especially this [learning and adapting] capability. A digital leader should simply provide the space for learning as well as leave room for continuous improvements. In the end, he or she should enable the own willingness to learn [from the team].*” Moreover, many experts described that digital leaders should also perform a **democratic leadership** to grant employees the freedom to contribute with ideas and concepts. In line with this, both top- and mid-level managers argue that digital leaders need to move away from the traditional autocratic and authoritarian way of leading which is summarised by one top-level manager with: “t*he understanding of leadership in a sense of command-and-control, hierarchy, decision-making power or setting the workload of one single person does simply no longer works today.”* A more participative and collaborative working culture covered by a democratic leadership style is seen as influential regarding the DISC of learning and adapting as *“it [democratic leadership] allows someone to ask questions, make suggestions or even give feedback.”* **Servant leadership** was described by an expert as *“serving the organisation as if my role is not important but the collective I lead is important,”* however was not mentioned in connection to the DISCs. The missing link to DSICs also applies to the **situational leadership** which depicted by experts as the situational awareness of digital leaders, and which is reflected in adaptive behaviours towards employees. Situational leadership was described by one expert with: “*leadership is always somewhat adapted to the situation, how the environmental variables are, how the team constellation is. On the one hand, as a manager, I cannot let a team that’s out of line dance so freely. I have to do something about it. On the other hand, I can let teams that are self-organised and achieve very good results run well on their own.”*

#### 4.2.2. Desired roles for digital leaders to strategically align teams

The desired roles for digital leaders influence the DSICs of signalling, co-producing and orchestrating, as well as scaling and stretching (den Hertog et al., 2010). Interviewees reported that digital leaders should appear as **coach, mentor, and facilitator** which was argued by one top-level manager with: *“Digital leadership is strongly linked to a role model and coach for its team which plays a more significant role nowadays. Hence, topics such as, facilitating support, giving orientation, coaching, creating common goals and forming a team, are more relevant than ever before. It’s less of a decision-making or hierarchical aspect which is certainly always a component. It’s more about challenging and encouraging employees – supporting them, coaching them, giving them a sense of purpose, and bringing these aspects to the fore.”* This role has an influence on the DSICs of signalling and scaling and stretching. Regarding signalling, a coaching, mentoring, and facilitating role of digital leaders helps to understand customer needs and to identify fitting technological options. In this sense, digital leaders rather accompany the team and the customer with their experience to make sure that processes and methods are applied correctly rather than solely deciding on a solution themselves. Given the DSIC of scaling and stretching, the experts underlined that digital leaders should coach, mentor, and facilitate related processes which owns a high grade of complexity, and which can be managed by decentralising functions and responsibilities. In this sense, a team demands a leader who empowers their efforts and accompanies the process with practical experience. One expert described this as follows: “*I heard a great saying the other day that you are only a good digital leader if you manage to get the people working for you to go beyond their limits and do the best they can and maybe even become better than you. Therefore, empowering is one of the fundamentals considering scaling. People can get the chance to freely develop frameworks and think outside the box.”* Experts further described the **guardrailer** role (i.e., jointly aligning on guardrails with teams) of a digital leader as pertinent in DT contexts, however with differing interpretations. One top-level manager mentioned that the guardrails to set are linked to *“establish framework conditions when it comes to workplace flexibility or working ours.”* Another top-level manager added that *“guardrailing goes beyond deciding on mere working structures for employees but establishes a whole new framework considering organisational structures in which the team is free to make own decisions, decides on parts of the budget and reviews development.”* In a similar vein, a mid-level manager reported on her experiences: *“The guardrailing capability of a digital leader is linked to set a clear framework for the team defining how to work together. This means that with the help of putting up a framework the goals or key results,* e.g.*, of a project, can be defined together as 60% of the goals and result comes from the team. That works wonderful with us because the team usually knows much better than the manager how the operative side works. But as a manager, I still have to set the framework for it and maintain it.”* Interview data reveal that digital leaders who appear as guardrailers positively influence the DSICs of signalling, and co-producting and orchestrating. To successfully identify service innovation potentials at an early stage (signalling), digital leaders need to align on common terms with the team and customers (e.g., not changing corporate designs). However, within these limits, all stakeholders must possess the opportunity to create and think freely about the user’s needs and fitting technologies. During co-production and orchestration, in which work on a service innovation overcomes organisational boundaries, experts agreed that digital leaders should set guardrails of where and how collaboration can happen. One top-level manager explained the co-production and orchestrating process as follows: “*When the application was developed, everyone [the customer and internal team] was involved and the project was embedded relatively high up in the hierarchy. Thus, the overall goal as well as direction was set from the beginning on. The teams, regardless of the hierarchy were completely free to choose how to achieve the final goal, however predefined guardrails, for instance target group of the app, had to be adhered.”* Data revealed that digital leaders are regarded as **sense-givers and navigators** by providing orientation towards teams, encouraging, and aligning with them on agreed-on goals or relating the company vision to the efforts of teams. One interviewee specified that *“[…] for me it’s more to put in the foreground encouraging and challenging my employees to give them a sense of purpose. I want to move slightly away from mere decision-making aspects; however, they certainly will be a component, and bring such issues to the fore.”* Experts further stated that appearing as a sense-giver and navigator influences the signalling DSIC. In this sense, one expert outlined that asking their teams the “right” questions help them to navigate through the abundance of available data to identify user needs and suitable technological solutions. By knowing the sense behind the customer’s purpose, the business mission, e.g., what do we want to achieve, which technological requirements does the customer have, can be derived. In this way, the team ultimately only proposes innovations to the customer that make sense as they fit the portfolio and are also achievable within the scope of the resources. Given the expert’s statements, digital leaders should take the role of a **people manager** in terms of sensing individual needs of employees and customers and addressing these needs proactively. In context of a hybrid working setup, this role was well described by one expert with: *“We’re going to end up in a hybrid world, we are not going to become fully office or fully remote - figuring out what my employees’ needs are at the point and responding to them individually because I cannot set the rule on what’s going to be gone after.”* In this way, another expert mentioned balancing employee and customer needs as a challenge for digital leaders: *“I have to bring together the customer needs and I have to bring together the employee needs. Yes, it can be more work for a manager to do this, and the pressure or the demands on managers from this - let us say - tactful logic are certainly increasing.”* Although people management was considered relevant by experts, no influence on managing technology-driven change in the DSICs context could be identified. This also applies for digital leaders acting as a **change agent** who *“must be able to get employees to work for the company’s interests in the best possible way.”* To achieve organisational change, experts mentioned approaches such as forming “coalitions of the willing,” receiving top-management commitment for transformation initiatives, broadly communicating the vision and strategy, and driving the development of strategic skills.

### 4.3. Organisational capital

Referring to the data structure, digital leaders take a pivotal role to foster new collaboration and structural approaches for an enhanced organisational agility. In terms of collaboration approaches, experts deemed the fostering of participation, result orientation, and continuous learning and feedback as relevant to influence DSICs. In this way, we evaluate a strong influence of collaboration approaches on signalling user needs and technological options, and a moderate influence on conceptualising, co-producing and orchestrating, and learning and adapting. Given structural approaches digital leaders may perform, experts assessed flat hierarchies, agile methods, digital tools, and digital working models as pertinent in the DSICs context. Structural approaches own a moderate influence on co-producing and orchestrating as well as on learning and adapting.

#### 4.3.1. Collaboration approaches digital leaders foster to drive organisational agility

Collaboration approaches affect the DSICs of signalling, conceptualising, co-producing and orchestrating, as well as learning and adapting (den Hertog et al., 2010). As observed in the data, **fostering participation** is relevant in DT contexts, notably commented by one top-manager *“From a traditional leadership point of view, we definitely developed to a more digital organisation faster than expected because of the topic of virtual collaboration and participation. Especially the interactions between managers and colleagues within the operational area. Of course, this is also reflected in participation due to the fact that you give up a little bit of your leadership role, in the sense of procedural control. Meaning that I do not have to lead through every meeting, even if I am the head of the department.”* Adding to that, one expert emphasized that *“as in the past, the leader said how it was done which is still valid for a digital leader, however, nowadays the focus shifted more to communicating what and why the team actually needs to do something in order to achieve a certain goal. Hence, it must further be specified how to get from start to beginning.”* The focus on participation is evident in three DSICs. First, given the DSIC of conceptualisation, the experts stated that a fluent participation in ideating and developing a service innovation concept is vital. In this sense, digital leaders need to closely work together with the customer and their team to agree on aspects such as pricing or target audience definition. Empowering both the team and customers was often described as a success factor outlined by one expert “*The most important term [during conceptualizing a service innovation] for me is empowerment. Because if you manage to empower your team to get themselves to innovate, then you actually automatically innovate and move forward.*” Second, the co-production and orchestration capability aims to manage service innovations across organisational boundaries. To achieve this highly participative setup, interviewees mentioned that digital leaders need to enable the working process of interdisciplinary teams. Frequently mentioned components of the co-production capability included the facilitation of transparent, eye-level communication and the invitation of all stakeholders to contribute on equal levels. Finally, for the learning and adapting capability, experts noted that digital leaders allow for ideas and opinions to reflect on them from the beginning on. This means that a service innovation can continuously be enhanced by empowering the team and customers to collaboratively give feedback. Most experts further reported that the principle of **aiming for result-orientation** is essential for the management of team performance. In this sense, one mid-level manager stated that *“I do not care not care how and when my employees do their work because I am interested which results are achieved at the end.”* Result-orientation also influences the three DSICs mentioned in collaboration and participation. According to experts, result-orientation helps digital leaders during conceptualisation to achieve a common understanding of the outcome of a service idea. The experts further noted that given the co-production and orchestration capability, communicating clear result expectations and responsibilities is fundamental as the service innovation is often times managed across organisational boundaries with a multitude of stakeholders involved. At the same time, experts note that digital leaders should consider result-orientation given the learning and adapting capability. Hence, in defining the specifications of the service innovation (e.g., prices or audience), the integration of learning and feedback cycles can be efficient. In this regard, one expert described that he beforehand conducts a workshop with the team and the customer to agree on how to transform ideas into a service innovation, while actively seeking feedback afterwards and customising the plan. Another expert extended this argument with: *“The customer and the team must understand what they can learn from an error which has been made and how they can avoid it the next time. To successfully innovate services, digital leaders need the courage to tolerate mistakes and to stand up for a trial-and-error culture because this is how a unique idea becomes an innovation.”* Moreover, interviewed experts underlined the importance of **enhancing continuous learning and feedback** for digital leaders. This principle addresses the capability to accept feedback about oneself or a project and learn from it for future progress which was stressed by one expert: *“On the one hand, we learn and get feedback from planned retrospectives. It is our aim that all concerns from the team arise during these retrospectives. So, in the sense we ask ourselves and others what we can learn from this or how we can avoid a certain situation in the future. On the other hand, these mechanisms are adapted to facilitate harm free experiments for the teams. In terms of the value system and culture of our organisation, it is important to allow employees to try innovative or digital things to see whether they are a success for the whole company.”* This principle influences the DSICs of signalling and conceptualising. To foster the signalling of user needs and technological options, one expert elaborated on two key criteria a digital leader needs to possess in contrary to a traditional leader. First, they need to allow their team to experiment and learn together with the customer. Second, being open in the sense of sharing knowledge openly with the team is also supportive to identify customer-fitting solutions and receiving feedback on it. Regarding the conceptualisation capability, several experts explained that when they lead the transformation process of a service idea into an actual offering, they actively seek feedback along all stages from the team and the client. In this case, experts described that they firstly approach the team with a new idea to get feedback in terms of feasibility, market, audience, and viability, and request customer feedback in a second step to test the desirability of the potential offering. Referring to the data, **developing organisational networking** becomes especially relevant in case a project requires specialist knowledge. In this context, one mid-level manager noted: *“When I deal with digital issues in my department, I increasingly need a specialist, for example, for cloud computing or digital infrastructure. And while in the past managers might have been still able to assess all areas themselves that is no longer possible today for a digital leader owing to the fact that the detailed knowledge for the individual area is much greater which changes the way we interact.”* Considering **improving self-organisation**, experts commented that digital leaders need to facilitate self-organised teams, among others by jointly defining goals. Data from the interviews did not reveal evidence for the influence of intra-organisational networking and self-organisation on any DSICs.

#### 4.3.2. Structural approaches digital leaders exhibit to develop organisational agility

Experts reported that structural approaches own an influence on the DSICs of co-producing and orchestrating, and learning and adapting (den Hertog et al., 2010). Experts supported digital leaders’ responsibility to drive the **flattening of organisational hierarchies**, however their opinions regarding the dimension of flat hierarchies differed. One top manager from a service organisation stated: *“I would like to add here that our company is organised in flat hierarchies meaning that our employees and managers together decide on outcomes and how they are achievable. This supports to implement self-efficacy and empowerment on all levels.”* In contrary, another top manager from the manufacturing industry underlined that a difference between organisations might come into play and explained that *“flat hierarchies within a department work fairly well, however, I do not think that flat hierarchies within a whole organisation are serving the goal, and hence, a grading from top to bottom and from bottom to top should be in place.”* Experts stated that digital leaders embracing the flattening of organisational hierarchies may enhance the DSIC of co-producing and orchestrating. In this sense, frequently mentioned approaches are the setup of transparent communication structures and the establishment of interdisciplinary teams. This leads to the transition from strict hierarchical decision-making procedures to communicating and discussing results with all stakeholders which fosters the managing of the service innovations across organisational boundaries as self-efficacy, self-organisation, and empowerment within teams and in relation with customers is increased. Furthermore, the interviews demonstrated that **developing agile working models** by digital leaders is crucial in DT contexts. One mid-level manager commented on agile methods as follows: *“It means that the manager provides an organisational framework and adheres to it. This can be, for example, an agile way of working, such as OKR methodology [objective and key result] or SCRUM, where the manager simply pays close attention to ensuring that the adopted frameworks are adhered to. Because flexibility, speed and self-organisation do not come from leading laissez faire. That is something completely different.”* Another manager added to that aspect that *“given digitalisation the team is becoming a bit more independent, hence self-responsibility is emerging now. Here, agile working methods are very helpful in bringing more responsibility into the teams. Enabling that is efficient digital leadership from my point of view.”* According to the experts, agile methods positively influence the co-producing and orchestrating of service innovations when facilitated by digital leaders. As this capability is associated with working in organisational networks, agile methods foster the assumption of responsibility emphasised by one expert with *“Agile ways of working are extremely helpful in bringing more responsibility to teams.”* In parallel, we found an influence of **fostering digital collaboration setups** on the DSIC of co-producing and orchestrating. By successfully managing service innovations in an inter-organisational setting, both top- and mid-level managers confirmed that digital leaders must support the application of digital tools throughout this capability. This creates more flexibility on intra- and inter-organisational levels which was described by one expert: “*When working in an inter-organisational setting, it is especially important to use digital tools. However, I have to use digital tools in such a way that, in principle, they also enable employees and people to work productively.”* As, fostering digital working setups are seen as crucial for digital leaders, experts also linked the topics of home office or remote work to it. One manager from a consulting organisation explained: *“The topic of home office is high on our agenda in connection with the pandemic and the respective change in working hours. We previously relied on a model with core working hours and have now switched to a fully flexible working model given our digital tools.”* Another manager described their handling of digital working models and setups with: *“We have decided not to adopt a working model regulation uniformly. We are building on activity-based working here. This means that when you come into the office, there really is something to do there. This means that you can work individually in the way that best suits your team. If you have to work creatively in a team or have a physical meeting, you meet in the office, but if you have an analysis task that requires rest, you stay at home and work remotely. Depending on what I want to do, I should choose the respective workplaces.”* Digital working models and setups were mentioned by experts considering the DSIC of learning and adapting. This can be reasoned with the decentralised and flexible character of modern working environments, especially fostered by the COVID-19 pandemic with the absence of physical meetings. This was expressed by one expert with regard to the design of hybrid working setups: *“When do we come together and when not? It should not be something like 50 percent of the week we are in the office or something like that, but it has to be somehow purpose-driven. So, we should come together for certain things and for other things we do not need to come together. And that has to be organized. That means the randomness of our collaboration will decrease.”* Given the DSIC of learning and adapting, experts suggested that digital leaders should flexibly assess when employees are in the need of physical meetings to creatively work on a service innovation.

## 5. Discussion

So far, empirical knowledge on leadership capabilities relevant to manage DT is scarce (Cortellazzo et al., 2019). Linked to the practical side, organisations still struggle to successfully embark their DT journey by gaining strategic value from acquiring technologies and digital knowledge (Schiuma et al., 2022). Among others, this can be attributed to the lacking people dimension which negatively affects business performance (Kotter, 2009; Deloitte, 2017; Davenport and Westerman, 2018). The present study follows emergent calls from academia to explore the leadership side of digitally transforming organisations (Avolio et al., 2014; Soto Setzke et al., 2021), whereas the service innovation context (Johne and Storey, 1998; Salunke et al., 2019; Gustafsson et al., 2020) represents an attractive opportunity to operationalise the analysis of DL-related capabilities. By answering how DL-related capabilities influence the management of technology-driven change over leveraging service innovations, this study especially extends the current knowledge in the DL research community and is also relevant in practical outlets.

### 5.1. Theoretical contributions

#### 5.1.1. Conceptualising digital leadership

Our examination of DL-related capabilities across a wide range of German service and manufacturing firms contributes to the still deficient conceptualisation of DL (Benitez et al., 2022) indicated by divergent understandings of the term and related capabilities across research streams. This fragmented picture is problematic as DT represents a business imperative for today’s organisations to achieve strategic change (Schiuma et al., 2022). For this reason, we aimed to make sense of DL-related capabilities by drawing on top- and mid-level manager’s knowledge and experience in DT projects (Gioia et al., 2013). Throughout our qualitative analysis, we discovered that DL-related capabilities address multiple dimensions in the form of a digital leader’s personal (characteristics and skills), social (behaviours and roles), and organisational capital (collaboration and structural approaches) which are differently assessed by experts regarding their relevance in DT contexts. Multi-level perspectives were also addressed by Eberl and Drews (2021) on the personal, individual (leader-follower interactions) and organisational level, and in research towards human-related capabilities in digital contexts such as Big Data analytics by incorporating the personal and organisational level (Korherr and Kanbach, 2021). Leadership theory refers to multi-level views as the movement from the person level to higher levels such as by linking leaders and followers on the individual, dyadic or group level, or towards macro-levels given by organisations, communities, and societies (Yammarino and Dansereau, 2008). In this vein, a leader is also responsible to optimise linkages between several levels, e.g., by formulating organisational strategies and ensuring their implementation on the micro-level (Mueller et al., 2020). We follow this view and propose that levels addressed by DL-related capabilities should be considered in an integrated way. As an example, a digital leader should focus on strategic thinking and delivery (personal capital) which links to leadership behaviours (e.g., transformational leadership) and roles (such as being a sense-giver and navigator) headed towards transforming an organisation by interacting with individuals and teams (social capital). This social capital should further be accompanied by the organisational capital of a digital leader to adjust collaboration and structural dimensions serving as preconditions for greater organisational agility such as by flattening hierarchies or by developing agile working models. This intertwined view resembles the concept of transcendent leadership firstly described in the field of strategic leadership (Crossan et al., 2008; Samimi et al., 2022). Using this study, we bring up this concept as a new perspective for the context of DL. Transcendent leadership argues that in dynamically changing settings such as given by DT, leaders need to tightly integrate the human-related levels of leading self (e.g., being self-aware and develop individual skills) and others (mechanisms to influence followers), but also continuously aligning non-human elements on an organisational level (environment, strategy, and the organisation). For this purpose, transcendent leadership incorporates different leadership approaches such as transformational, transactional, charismatic, shared, and authentic leadership (Crossan et al., 2008). Bringing these perspectives together, we contribute to the level discussion in leadership research, traditionally anchored in micro-oriented perspectives (Crossan et al., 2008; Mueller et al., 2020), towards a more holistic and integrated view of DL.

With regards to the blending of traditional and modern leadership capabilities (Kane et al., 2019), we identified several capabilities which we consider as distinctive for a digital leader to drive and manage DT-related change. These capabilities include adaptability (personality characteristics); digital literacy, strategic thinking and delivery, and diverse team leadership (leadership skills); transformational and situational leadership behaviours (leadership behaviours); coach, mentor and facilitator, guardrailer, sense-giver and navigator (leadership roles); enhancing organisational networking and self-organisation (collaboration approaches); and driving agile working models as wells as digital collaboration setups (structural approaches). Apart from expert nominations, which partly result in diverging DL-related capabilities in terms of their perceived relevance (see Figure 2), we reason this selection with two macro trends identified in DL literature. First, an increased grade of relationship-oriented leadership in digital business setups exists indicated by a coaching and enabling leadership behaviour, an individualised consideration of employee needs, an increased networking behaviour, and the focus on building dynamic and virtual teams (Schwarzmüller et al., 2018). Referring to the taxonomy of DL-related capabilities, the elevated relationship orientation is reflected in the personal capital of a digital leader such as by diverse team leadership skills, in the social capital regarding transformational and situational leadership behaviours and the roles of a coach, mentor and facilitator, sense-giver and navigator, and finally in the organisational capital manifested in fostering organisational networking and digital collaboration setups. Another macro trend denotes the strategic character of DL (Westerman and McAfee, 2012; Kane et al., 2019; Petry, 2019; Benitez et al., 2022; Erhan et al., 2022) which is represented by a digital leader’s personal capital such as adaptability, digital literacy, strategic thinking and delivery, by the social capital with transformational leadership behaviour and the role of a guardrailer, and sense-giver and navigator, and by organisational capital concerning the development of self-organisation and agile working models in organisations. This proposition extends the view from Kane et al. (2019) who consider a transformative vision and forward-looking perspective, digital literacy, and adaptability as distinctive while enact capabilities associated with the articulation of change and the commitment of resources towards it, the leadership of an organisation’s digital transformation, and the enablement and empowerment of employees as non-distinctive for a digital leader. Overall, our taxonomy of DL-related capabilities shows several overlaps with literature (see Table 2), especially in terms of a digital leader’s personal (e.g., adaptability, openness, digital literacy, strategic thinking, communication and remote leadership skills) and social capital (e.g., transformational and democratic leadership behaviour, and acting as a coach, mentor, sense-giver and navigator) which support the conceptualisation efforts for DL.

Regarding the personal capital of a digital leader, literature among others discusses the breadth and depth of digital skills (Fisk, 2002; Schwarzmüller et al., 2018; Kane et al., 2019; Petry, 2019; Klus and Müller, 2021; Gilli et al., 2022). In this sense, our data point to digital leaders possessing a *“general digital literacy”* (Kane et al., 2019) in terms of a solid digital understanding which may diverge based on the thematic focus of organisational units and the type of business model. This view opposes propositions by Fisk (2002) describing digital leaders as *“techno-savants”* with a deep technological expertise. Based on our findings, we understand “digital literacy” as a leader’s digital mindset, underlined by principles such as digitally leading by example, transparency, collaborative thinking and skill diversity, continuous learning and experimentation, and customer centricity; strategic skills to continuously develop the digital portfolio and the skill base consistent to the business model; data management skills (e.g., by combining data from multiple sources) to foster data-based decision making; and digital collaboration and workflow skills to provide optimal conditions for team productivity and cohesion.

An often-discussed aspect in DL research is the impact question of leadership behaviours such as towards innovative work behaviour, creativity, and team effectiveness (Wu and Cormican, 2020; Erhan et al., 2022; Zhu et al., 2022). The range of behaviours identified in our data (transformational, democratic, servant, situational) underlines the behavioural flexibility digital leaders need to present in DT contexts. This implies a departure from pure transactional leadership approaches towards a blending of leadership behaviours which goes in line with employee- and situation-specific leadership behaviours many of the interviewed experts revealed, and which can be embedded into the contingency theory of leadership (Fiedler, 1964). Our results further reveal that behavioural ambiguity is also reflected in a digital leader’s roles (coach, mentor, and facilitator; guardrailer; sense-giver and navigator; people manager; change agent). In correspondence to Weber et al. (2022), these roles map to task-oriented (e.g., digital pioneer and innovator) and people-oriented (e.g., enabler and mentor) roles digital leaders should exhibit. Surprisingly, although ambidextrous leadership behaviour is considered as relevant to DL (Petry, 2019; Benitez et al., 2022), we did not find evidence in our data in the sense of an explicit naming of the term or synonyms.

As the emphasis of leadership research is traditionally anchored in characteristics, skills and behaviours (Crossan et al., 2008; Cortellazzo et al., 2019), incorporating a digital leader’s organisational capital helps to advance DL research by identifying key responsibilities of a digital leader dedicated towards the firm level to guide an organisation in dynamic environments shaped by DT. Our results regarding a digital leader’s organisational capital are thematically consistent with literature (see: “Functions” in Table 2). This applies to the development of an organisation’s collaboration culture, such as by enhancing organisational networking, self-organisation, and participation (Schwarzmüller et al., 2018; Kane et al., 2019) and the redesign of organisational structures towards more agile and digital ones (Fisk, 2002; Petry, 2019; Benitez et al., 2022). In this vein, literature mentions the alignment of business and IT entities, and the establishment of governance structures (Westerman and McAfee, 2012; Buvat et al., 2018) which, however, our data do not detail. The development of a digital strategy and vision (El Sawy et al., 2016; Kane et al., 2019; Petry, 2019) as well as the attraction and development of talent (Schwarzmüller et al., 2018; Kane et al., 2019; Benitez et al., 2022) represent further functional aspects of a digital leader prominently addressed in literature, which are covered by our taxonomy *via* the roles of a sense-giver and navigator, and change agent.

In summary the central contribution of our taxonomy is its holistic character which fosters a uniform understanding of DL-related capabilities beyond the micro-oriented research discourse in DL (Eberl and Drews, 2021) by encompassing findings from IS and management research towards the organisational level. This is supported by Weber et al. (2022) who argue that although leadership is crucial for DT-related company-wide change, knowledge regarding the integration of both levels is still scarce.

#### 5.1.2. Developing DL capabilities to manage digital transformation in a service innovation context

To explore DL’s influence on the management of digital transformation, we took a micro-foundational perspective on dynamic capabilities by studying DL-related capabilities and connecting them to the DSICs framework. The DSICs framework validated by Janssen et al. (2016) serves as a reference for service and manufacturing firms to establish a competitive advantage by the ability to continuously deliver service innovations (den Hertog et al., 2010). Thus, we consider the intersection of DL-related capabilities and DSICs as an attractive research opportunity which, to the best of our knowledge, represents a novel research perspective on the human-related side of digital transformation. For this purpose, we applied a qualitative research approach which answers research questions about the “why” and “how” of a novel phenomenon (Pratt, 2009; Bluhm et al., 2011; Langley and Abdallah, 2015). In this way, a qualitative investigation of influences of DL on DSICs is consistent with related research on DT applying the DCV (Soluk and Kammerlander, 2021).

Our conceptual framework reveals that a digital leader’s personal and organisational capital owns several strong and moderate influences on DSICs, and hence, is most relevant for digital leaders to manage technology-driven change in a service innovation context. Although the current academic discourse on leadership behaviours identifies a positive influence on organisational (Alblooshi et al., 2021) and especially on service innovation (McAdam and Mitchell, 2010; Michaelis et al., 2010; Kao et al., 2015), our study assesses the influence of a digital leader’s social capital on DSICs (signalling user needs and technological options, co-producing and orchestrating, scaling and stretching, learning and adapting) only as weak. We argue the weak influence of social capital and the strong influence of personal and organisational capital with the character of the DSICs framework (den Hertog et al., 2010). DSICs own an interdisciplinary (multiple organisational units involved) and cross-organisational nature requiring a highly collaborative setup. This setup is shaped by a digital leader’s characteristics and skills, and leadership interventions towards the cultural and structural development of an organisation. In this sense, as digital leadership behaviours such as given by the context of ambidextrous leadership unfold over micro-levels (Mueller et al., 2020), their transformative effects may show indirectly over the interaction with followers. This argumentation follows service research stating that leading service innovation projects usually depends on a micro-level to achieve organisational impact rather than enclosing an organisation-wide perspective (Eisingerich et al., 2009). Further, literature on DL argues that digital leaders need a composite of characteristics (Petry, 2019; de Araujo et al., 2021; Benitez et al., 2022) and skills to foster innovation performance such as by positively influencing innovative work behaviours (Erhan et al., 2022) or by digitizing a firm’s platform which further impacts organisational innovativeness (Benitez et al., 2022).

Overall, our framework reveals unequally distributed influences of DL-related capabilities pointing towards the characteristic of DSICs to independently contribute to the management of digital transformation. This goes in line with Janssen et al. (2016) who empirically confirmed that firms from the service and manufacturing industry can obtain a competitive advantage by implementing service innovations without stringently applying all DSICs in conjunction. Our findings contribute to the dynamic capabilities literature (Teece, 2007) by identifying DL-related capabilities and by showing links towards several DSICs relevant to manage DT. This represents a new perspective to the microfoundations discussion elucidating how dynamic capabilities (sensing, seizing, transforming) evolve from the interplay of managerial and organisational microfoundations (Bendig et al., 2018). Although literature has indicated that DL plays a significant role in digitally transforming companies within the service innovation context, e.g., by developing adaptive learning cultures (Anning-Dorson, 2016; Verdu-Jover et al., 2018) or by building up an organisation’s innovation capacity (Singh et al., 2020; Soomro et al., 2021), this intersection remains a gap our results contribute to with an enriched capability understanding.

### 5.2. Managerial contributions

As firms often struggle to realise the full potential of DT initiatives (Fitzgerald et al., 2014), this study also poses a valuable contribution for practitioners in three respects.

First, managers can refer to the taxonomy as a comprehensive tool to assess and improve the status quo of their DL-related capabilities. As such, the structure provides executives orientation to select and develop their leadership personnel (Cascio and Aguinis, 2008; Day et al., 2014), whereas the multi-level character sensitises decision-makers about the broadness of capabilities digital leaders must possess in DT contexts. Second, building up DL-related capabilities fosters the development of organisational cultures towards more digital ones which is seen as crucial in a DT context (Fisk, 2002; Kane et al., 2019). Digital cultures are reflected by attributes such as a digital mindset, innovation culture, agility and flexibility, shared and data-driven decision processes, employee engagement, and boundary-less collaboration (Fisk, 2002; Patterson et al., 2005; Petry, 2019). Third, referring to the conceptual framework, managers can now pinpoint to determinants (DL-related capabilities) of service innovation management which drives the DT within their organisation. Hence, from their current organisational state, they can build up DL-related capabilities having a strong influence on service innovation projects. We consider this as crucial for digital leaders as they take an instrumental role in the innovation process and organisational change (Denti and Hemlin, 2012), especially in the context of DT (Jian Zhu et al., 2022).

### 5.3. Limitations and further avenues for research

The results of this study show that DL-related capabilities are determinants of the management of DT given a service innovation context. Apart from theoretical and practical contributions, this paper has limitations leading to further research avenues.

Referring to aspects of sampling, the study’s scope refers to the broad field of German service and manufacturing organisations which formed the basis of our analysis. Although the field of service innovations can be regarded as transdisciplinary (Aksoy et al., 2020) supporting the broadness of our sample, we did not differentiate regarding the size of organisations or legal forms. Moreover, data collection was conducted only with top-and mid-level managers from the above-mentioned organisations. At this point, future research can pick up and validate results against the backdrop of other stakeholders such as customers, business partners or employees. Besides, future research can study the influence of DL-related capabilities on service innovations in a more nuanced way such as by only sampling digital-savvy firms (e.g., software companies). An exemplary research topic in this context may be the study of the influence of a digital leader’s behaviours on DSICs depending on the service type (digital vs. non-digital) and scale. As the data was collected during the pandemic situation, further research can also examine whether results of this study have changed and assess whether DL-related capabilities are still valid in this regard.

Another limitation is connected to the exploratory character of our study applying a qualitative methodology. This means that the proposed relations between DL-related capabilities and DSICs are conceptual in their nature and were not quantitatively validated. This also includes an outcome perspective of DSICs which we descoped for this study. In this way, future research may deliver a statistical model which validates interdependencies and effects of DL-related capabilities on DSICs as well as their outcomes indicating a firm’s competitive advantage.

To clarify the multi-level character of DL, further qualitative research designs such as case studies may help to illuminate the process of how technology-driven change unfolds through the interplay of DL-related capabilities. Insights about the transcendental character of DL may serve as a role model for organisations of how exactly to achieve a competitive edge.

As we characterise DL as an umbrella term, specifying overlaps and disjunctions from other leadership theories and models further cultivates the conceptualisation of DL. This especially applies to agile leadership also prominently mentioned in practical contexts (Parker et al., 2015; Fachrunnisa et al., 2020; Langholf and Wilkens, 2021) as well as e-leadership (Avolio et al., 2014) and Digital Transformation leadership (McCarthy et al., 2021) oftentimes described synonymously to DL in theoretical contexts.

## Data availability statement

The datasets presented in this article are not readily available because of confidentiality agreements with our interview partners. Requests to access the datasets should be directed to the corresponding author.

## Author contributions

TB, TS, and CL contributed to conception and design of the study. TB and TS organised the database, performed the qualitative analysis, wrote the first draft of the manuscript and implemented feedback given by CL. CL supervised the process. All authors contributed to the article and approved the submitted version.

## Conflict of interest

The authors declare that the research was conducted in the absence of any commercial or financial relationships that could be construed as a potential conflict of interest.

## Publisher’s note

All claims expressed in this article are solely those of the authors and do not necessarily represent those of their affiliated organizations, or those of the publisher, the editors and the reviewers. Any product that may be evaluated in this article, or claim that may be made by its manufacturer, is not guaranteed or endorsed by the publisher.
